# Syndecan-1 Is Required to Maintain Intradermal Fat and Prevent Cold Stress

**DOI:** 10.1371/journal.pgen.1004514

**Published:** 2014-08-07

**Authors:** Ildiko Kasza, Yewseok Suh, Damian Wollny, Rod J. Clark, Avtar Roopra, Ricki J. Colman, Ormond A. MacDougald, Timothy A. Shedd, David W. Nelson, Mei-I Yen, Chi-Liang Eric Yen, Caroline M. Alexander

**Affiliations:** 1 McArdle Laboratory for Cancer Research, University of Wisconsin-Madison, Madison, Wisconsin, United States of America; 2 Department of Dermatology, University of Wisconsin-Madison, Madison, Wisconsin, United States of America; 3 Department of Neuroscience, University of Wisconsin-Madison, Madison, Wisconsin, United States of America; 4 Wisconsin National Primate Research Center, Madison, Wisconsin, United States of America; 5 Department of Molecular & Integrative Physiology, University of Michigan, Ann Arbor, Michigan, United States of America; 6 Department of Mechanical Engineering, University of Wisconsin-Madison, Madison, Wisconsin, United States of America; 7 Department of Nutritional Sciences, University of Wisconsin-Madison, Madison, Wisconsin, United States of America; Stanford University School of Medicine, United States of America

## Abstract

Homeostatic temperature regulation is fundamental to mammalian physiology and is controlled by acute and chronic responses of local, endocrine and nervous regulators. Here, we report that loss of the heparan sulfate proteoglycan, syndecan-1, causes a profoundly depleted intradermal fat layer, which provides crucial thermogenic insulation for mammals. Mice without syndecan-1 enter torpor upon fasting and show multiple indicators of cold stress, including activation of the stress checkpoint p38α in brown adipose tissue, liver and lung. The metabolic phenotype in mutant mice, including reduced liver glycogen, is rescued by housing at thermoneutrality, suggesting that reduced insulation in cool temperatures underlies the observed phenotypes. We find that syndecan-1, which functions as a facultative lipoprotein uptake receptor, is required for adipocyte differentiation *in vitro*. Intradermal fat shows highly dynamic differentiation, continuously expanding and involuting in response to hair cycle and ambient temperature. This physiology probably confers a unique role for Sdc1 in this adipocyte sub-type. The PPARγ agonist rosiglitazone rescues *Sdc1−/−* intradermal adipose tissue, placing PPARγ downstream of Sdc1 in triggering adipocyte differentiation. Our study indicates that disruption of intradermal adipose tissue development results in cold stress and complex metabolic pathology.

## Author Summary

All mammals strive to maintain a fixed body temperature, and do so using a remarkable array of different strategies, which vary depending upon the degree of cold challenge. Physiologists many decades ago observed that a fat layer right underneath the epidermis (and above the dermal muscle layer) thickens in response to colder ambient temperatures. This “intradermal fat” provided insulation within days of climate changes. We have found that syndecan-1, which functions as a facultative lipoprotein uptake receptor, is required for intradermal fat expansion in response to cold exposure. This is a highly specific phenotype not shared by other adipocytes. When intradermal fat is absent, mice do not adapt normally to cold stress, and show altered systemic physiologies, including increased brown adipose tissue thermogenesis and hyper-activation of a stress checkpoint (p38α), designed to protect the body against mutagenic and oxidative stressors. The phenotypes associated with loss of Sdc1 function are reversed when mice are housed in warm temperatures, where defense of body temperature is not required. This study is the first to show that intradermal fat can be genetically regulated, with systemic effects on physiology.

## Introduction

Mammals have an extraordinary ability to defend their body temperature, and their homeothermy is supported by high calorie expenditure; indeed for mice, a transition from a warm, “thermoneutral” (30–33°C) temperature downward to the prescribed laboratory housing temperature (typically 20–24°C) increases the metabolic load by 50–60% [1], [2], [3], [4]. Metabolic mechanisms that promote efficiency are therefore key, especially for mice chronically housed under conditions that constitute (mild) cold stress. There is a well-established cascade of sensory and reactive components of non-shivering adaptive thermogenesis, often starting with cold-activated local and sympathetic neural response mechanisms [5], [6], [7], [8], although non-neural, cellular level mechanisms have also been described [9]. These sensors induce activation of both white and brown adipose tissues, to enable circulatory warming via oxidation of lipids [10]. Although physiologists have stressed the importance of insulation for many years, there are no studies that describe adaptive changes of skin/fur in mice housed in mild cold stress. Since the responses to cold stress clearly impact many processes, including macrophage activation [5], the immune response to tumorigenesis [11], and obesity [12], factors that mitigate cold stress are important to understand. Serendipitously, our studies of mice with a mutation in syndecan-1 (Sdc1) have revealed a role for this molecule in maintaining normal intradermal fat function and alleviating cold stress.

Syndecan-1 (Sdc1; CD138) is an abundant heparan sulfate proteoglycan that is expressed by most epithelial cells, and by stromal, endothelial and hematopoietic lineages during active phases of their development [13]. Its function is often dominated by its constituent heparan sulfate side chains, which are proposed to enable growth factor signaling by promoting ligand/receptor complex formation [14], [15]. Despite the implication of Sdc1 in the activity of a great many growth factors and cell adhesion molecules, *Sdc1−/−* mice are viable, fertile and grossly normal. Their only obvious phenotype is their smaller size; they have the same body composition as wild type mice, but are systematically smaller throughout growth and development by approximately 13% [16]. These mice do show highly significant phenotypes such as tumor resistance [16], [17], altered stress responses and wound healing, and changes in B cell development and microbial pathogenesis [18]. More recently, *Sdc1−/−* mice have been shown to have defects in lipoprotein particle metabolism [19], [20], leading to altered levels of circulating VLDL.

Here, we describe a general physiological change in *Sdc1−/−* mice that has the potential to influence a wide variety of stress responses, including tumor development. We show that these mice are cold stressed in normal housing conditions, and that this is associated with a deficiency of intradermal fat. Brown adipose tissue in *Sdc1−/−* mice shows elevated UCP1 and increased p38α activation, and this stress checkpoint is also more systemically activated in intraperitoneal organs. Furthermore, our experiments support a key function for Sdc1 as a VLDL receptor for undifferentiated adipocytes, and as an essential trigger for adipocyte differentiation *in vitro*. Expansion of intradermal fat in *Sdc1−/−* mice can be rescued by administration of the PPARγ agonist, rosiglitazone, *in vitro* and *in vivo*. We hypothesize that Sdc1 is required by intradermal fat, and not by white adipose tissue, because intradermal fat is constantly remodeled in response to both ambient temperature and the hair cycle. Our model proposes that inhibiting Sdc1 activity *in vivo* could induce p38α activation to modulate a number of physiologies.

## Results

### Calorie stores are depleted in *Sdc1−/−* mice

**Figure 1:**
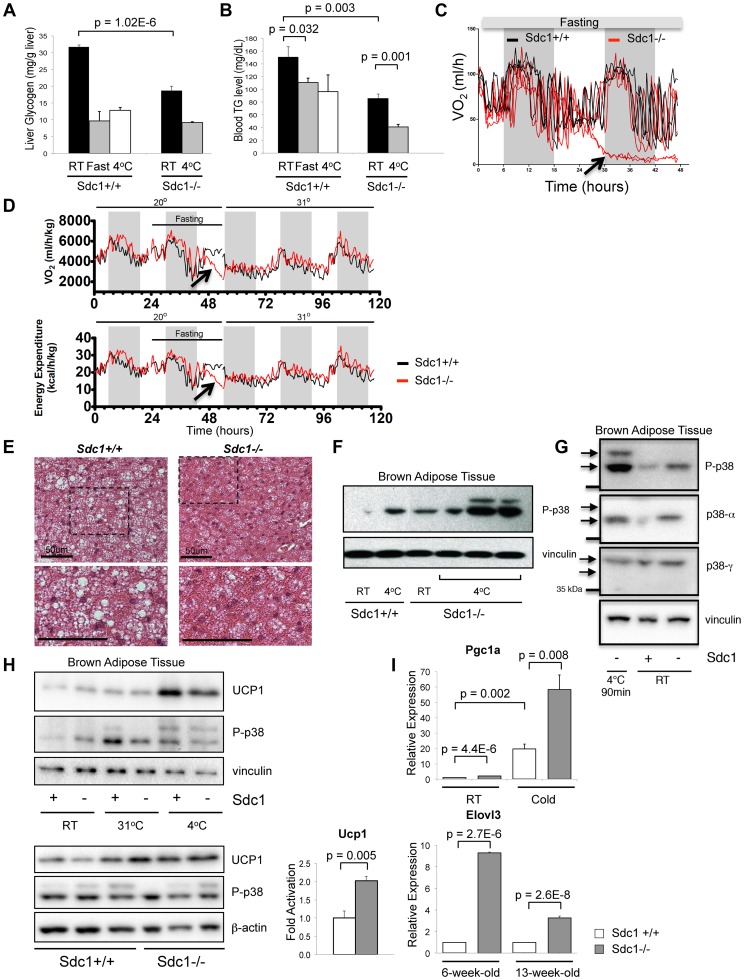
*Sdc1−/−* mice are chronically cold- stressed. **A**. Liver glycogen was measured for *Sdc1−/−* and wild type BALB/c mice under normal housing conditions (room temperature; RT; 21°C), after a 24-hour fast or after acute cold stress (90 mins at 4°C; n = 6). **B**. Serum triglycerides were measured for the same conditions. **C**. Mice of both genotypes were housed in metabolic cages at 23°C, fasted and their metabolic rates were measured (oxygen consumption, expressed as VO_2_). In this example, *Sdc1−/−* mice entered torpor after 24 hours (indicated as a precipitous drop in O_2_ consumption for two out of four *Sdc1−/−* mice in this experiment). None of the wild type (0/4) mice triggered torpor. **D**. In this example, measurements from 4 BALB/c (black lines) and 4 *Sdc1−/−* (red lines) mice were averaged (3/4 *Sdc1−/−* mice and 0/4 BALB/c mice entered torpor, indicated by the blue arrows). The housing temperature was shifted up to 31°C to illustrate their complete recovery (shown as VO_2_/oxygen consumption and energy expenditure curves over 3 days). **E**. Brown adipose tissues were harvested from *Sdc1−/−* and BALB/c mice housed at 21°C. H&E-stained paraffin sections show that *Sdc1−/−* brown adipose shows depleted lipid stores (white cytoplasmic inclusions). **F**. To test for cold stress, lysates of brown adipose tissue (shown here from three independent mice) were assayed for activation of the checkpoint, pT^180^Y^182^ p38α, either at room temperature or after acute cold stress (4°C). **G**. To confirm which isotypes of p38 were present and activated, the pattern of phospho-p38 was compared to Western blots probed with isotype-specific antibodies. **H**. Lysates were probed for UCP1, a marker of uncoupling in brown adipose tissue. Top panel, BAT from mice housed at various housing temperatures; bottom panel, assay of relative levels of UCP1 in *Sdc1−/−* mice housed at RT. **I**. To test whether the BAT of *Sdc1−/−* mice had initiated the full thermogenic program, levels of cold stress-induced mRNAs were measured by qPCR for tissues isolated from mice housed at RT and 4°C/cold (5 days) (PGC1α, n = 3) and for mice of different ages (6- and 13-week-old; Elovl3).

As part of our investigation of the tumor-resistant phenotype of *Sdc1−/−* mice, we analyzed baseline metabolic parameters, seeking systemic differences [16]. In general, liver-associated glycogen provides a carbohydrate buffer to meet short-term calorie needs, and the levels of circulating triglycerides reflect an intricate sum of fat uptake, mobilization and oxidation [21]. We evaluated the dynamic range of both these calorie reserves after challenge by two metabolic stressors, cold and fasting. BALB/c mice showed 70% reduced liver-associated glycogen after 90 minutes of 4°C cold exposure, or an overnight fast (Fig. 1A). Circulating triglycerides were reduced to approximately 100 mg/dL (Fig. 1B). We found that glycogen was depleted in livers from *Sdc1−/−* mice (by nearly 50%, from 32 to 19 mg/g liver, p = 1×10^−6^; Fig. 1A), and the circulating levels of triglycerides were not normal (also 50% reduced, from 151 to 86 mgs/dL, p = 0.003; Fig. 1B). These are broadly similar to the calorie depletion patterns observed in wild type BALB/c mice stressed by cold or fasting [22]. Cold stressed *Sdc1−/−* mice showed plasma levels of triglycerides of only 41 mgs/dL, which is out of the normal range.

### Sdc1−/− mice are susceptible to torpor and show chronically activated brown fat

During experiments designed to assay metabolic stress-related responses, we noticed that *Sdc1−/−* mice housed at room temperature (defined for this study as 20–23°C), were unusually prone to entering torpor after overnight fasting [23], [24]. Thus, assay of the metabolic activity of mice housed in metabolic cages at room temperature showed that some *Sdc1−/−* mice showed a sudden steep decline in O_2_ uptake after 24 hours of fasting, and entered a semi-comatose state (Fig. 1C, D). None of the wild type mice showed this behavior (p = 0.02, 2-sided Fisher exact). Consistent with other studies, this state of torpor was reversible by shifting the housing temperature to 31°C (Fig. 1D). Torpor is a conservative physiological response associated with decreased cardiac activity, and is typically observed in normal mice only in response to dual stressors, both cold and fasting [3], [24], [25]. This result suggested to us that *Sdc1−/−* mice were abnormally cold-stressed.

To test this hypothesis, we examined the main systemic modulator of temperature homeostasis, the brown adipose tissue (BAT), for evidence of activation of a thermogenic response. Brown adipose tissues are specialized to react to cold stress via a sensory mechanism that includes βadrenergic- and/or cardiac natriuretic peptide-dependent activation of mitogen activated protein kinase-14 (MAPK14)/p38α [26], [27], [28]. Activated p38α in BAT induces the uncoupling and biosynthesis of mitochondria, generating heat fueled by oxidizing local fat depots; the effect is to warm incoming blood to maintain thermal homeostasis [12], [29], [30].

We assayed BAT from *Sdc1−/−* mice, and observed that the lipid reserves were severely depleted (Fig. 1E). Chronic demands for heat generation are known to lead to calorie depletion of BAT and enhanced VLDL clearance [22]. Chronic cold stress is also known to sensitize mice and affect subsequent responses to acute stressors [31]. We therefore tested molecular markers of cold stress in BAT, and the response of these mice to acute cold (transfer to 4°C for 90 minutes). We assayed activation of p38α assayed as phospho-T^180^Y^182^ p38α) since this signaling hub is required for thermogenesis [26], [27], [32]. After cross-checking with isoform-specific antibodies, we showed that phospho-p38α was increased in BAT of *Sdc1−/−* mice housed at room temperatures, to levels approximately equivalent to that observed in BALB/c mice upon acute cold stress (Fig. 1F, G). Furthermore, *Sdc1−/−* mice showed super-activation after transfer to 4°C. Uncoupling protein-1, UCP1, is a heat-generating mitochondrial protein that is fundamental to the response of white and brown adipose tissues to cold stress [30], [33]. The expression of UCP1 protein was increased by 10-fold in the BAT of acutely cold-exposed mice (Fig. 1H). UCP1 protein levels were increased by 2.0-fold (p = 0.005) in BAT of *Sdc1−/−* mice in RT housing (Fig. 1H). We measured markers of transcriptional activation of BAT; PGC1α mRNA (Peroxisome proliferator-activated receptor gamma coactivator 1-alpha, *PPARGC1A*) is crucial for inducing mitochondrial proliferation and was elevated 2.0-fold in chronically housed *Sdc1−/−* mice and super-induced upon acute cold stress (Fig. 1I) [34], [35]. Elovl3 [36] was increased 3 to 9-fold in *Sdc1−/−* mice in RT housing, and has been associated with cold stress before.

### *Sdc1−/−* mice show deficient intradermal fat

**Figure 2:**
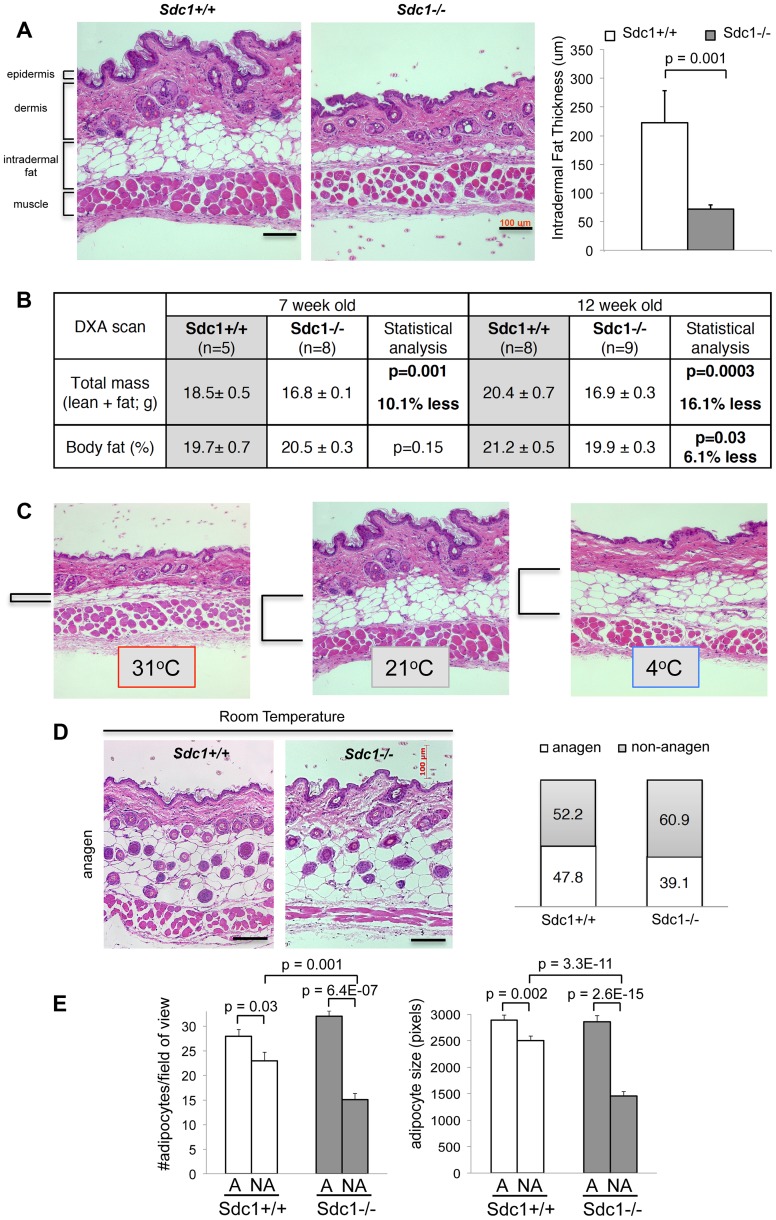
*Sdc1−/−* mice have thinner intradermal fat. **A**. Paraffin-embedded belly skin samples of *Sdc1−/−* and wild type BALB/c mice housed at 23°C were sectioned, H&E stained and the thickness of intradermal fat was quantified for non-anagen stage skin samples, measuring from muscle to dermis (n = 18 and 21 respectively). **B**. Body mass index was measured by DXA imaging for two groups of *Sdc1−/−* and wild type mice (as indicated). **C**. Paraffin-embedded belly skin samples of wild type BALB/c mice, housed at 31°C (5 days), 23°C (constant housing) and 4°C (5 days), were sectioned to illustrate the effect of housing temperature on intradermal fat deposits. Intradermal fat is indicated by brackets. (Back skin showed similar effects, but all skins compared for any one experiment come from the same location). **D**. Samples of anagen-stage belly skin for *Sdc1−/−* and wild type BALB/c mice housed at 23°C were stained with H&E. The hair follicle cycle is asynchronous in adults; anagen-stage is defined by the intrusion of hair follicles to the bottom of the intradermal fat layer, and a high epithelial mitotic index (Fig. S3). Skin samples were scored as anagen or non-anagen for cohorts of *Sdc1−/−* and wild type BALB/c mice, and the proportion of each is illustrated (right hand side), to provide an estimate of relative frequency of anagen-stage skin. **E**. Morphometric analysis of adipocytes shows that size and number of adipocytes is normal in *Sdc1−/−* skin during anagen, but both are reduced in non-anagen stage *Sdc1−/−* skins (n = 6).

We hypothesized that *Sdc1−/−* null mice perceive abnormal levels of cold stress in normal housing conditions, and searched for the underlying physiology. Since the reactive component of thermogenesis, brown adipose tissue, appeared to be functionally normal, we turned instead to a tissue that has been proposed to be key to thermal insulation, the intradermal layer of fat in the skin [37]. In *Sdc1−/−* skin, we found the intradermal fat layer was dramatically reduced, by at least 75% (Fig. 2A). The fat layer comprised of fewer, smaller adipocytes, measured histologically (Fig. 2E). All the other tissue types comprising the skin were normal in *Sdc1−/−* mice (epidermis including hair follicles, dermis and muscle; data not shown and Fig. S1, S2).

We considered the possibility that the deficiency of intradermal fat in *Sdc1−/−* mice reflected a generalized loss of white adipose tissue. We therefore measured fat content of *Sdc1−/−* mice using dual-energy X-ray absorptiometry (DXA) imaging [38], and found that the size of fat depots were no different for peri-pubescent *Sdc1−/−* mice (7 weeks old), with a minor (though statistically significant) decrease in body fat for 12 week-old mice (Fig. 2B). Note that *Sdc1−/−* mice are approximately 13% smaller than BALB/c wild type mice throughout embryogenesis and adulthood [16]. Measurements of individual fat pads (mammary glands and gonadal/peri-uterine fat pads) confirmed the DXA result (data not shown), and histological analysis of white adipose tissues showed that the size of adipocytes in white adipose tissue was not different in *Sdc1−/−* mice; neither were the expression of several molecular markers of white adipose cells (Fig. S3).

### Intradermal fat is regulated in response to ambient temperature and the stage of the hair cycle

Though the regulation of intradermal fat is almost entirely uncharacterized, it is known to expand during folliculogenesis to support the anagen phase of hair growth [39](data redrawn in Fig. S4). Indeed a recent report described the functional interaction of a mesenchymal cell type in the intradermal fat with folliculogenesis [40]. Furthermore, intradermal fat is directly responsive to ambient temperature. Thus when BALB/c mice are housed at 31°C for 2 weeks, intradermal fat thins out to only 20% of the thickness typical of normal housing temperatures (Fig. 2C). This “minimum” thickness corresponds to the thickness observed in *Sdc1−/−* mice housed at room temperatures. Indeed, it is known that mice housed at room temperatures are relatively cold-stressed [1], [4], [23], [41]. Intradermal fat depots may be particularly important for determining the physiology of mice housed between isothermal and room temperatures, since intradermal fat expansion shows a dynamic response for this temperature range (the additional cold stress imposed by housing at 4°C did not increase the intradermal fat layer; Fig. 2C). The intradermal adipocyte depot therefore responds to entirely different cues compared to other adipocyte depots.

When we examined anagen stage skin from *Sdc1−/−* mice, the intradermal fat layer was the same thickness as control mice (Fig. 2D), reflecting an equivalent number and size of adipocytes (Fig. 2E). Anagen stages comprise approximately 2 weeks out of 4 for each cycle period, and the proportion of samples from *Sdc1−/−* mice that were in non-anagen was approximately the same as control mice (Fig. 2D), suggesting that the follicle cycle time was not grossly affected by this mutation. (We define non-anagen as absence of follicular penetration below the dermis). Furthermore, neither the initiation of the first hair cycle (scored as the gross appearance of hair at day 6), nor the follicular density during anagen was affected by the absence of Sdc1 (Fig. 2D, S1) [40]. We conclude that the effect of Sdc1 is specific to intradermal fat and not other adipocyte reserves, and that it is important only to the expansion of adipocytes in response to ambient temperatures, and not to the local signaling that regulates hypertrophy during anagen.

### Sdc1−/− mice show systemic activation of the p38α stress checkpoint

**Figure 3:**
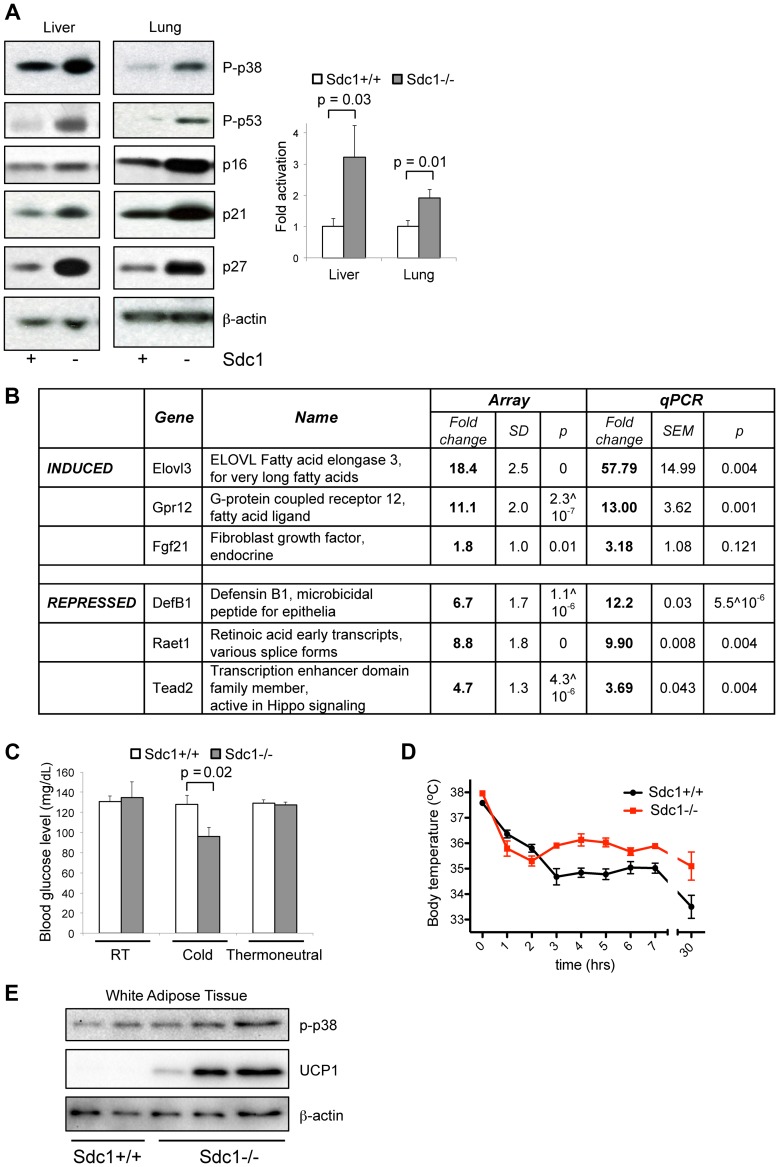
*Sdc1−/−* mice show systemic hyper-activation of p38α, and other metabolic markers of cold stress. **A**. Tissue lysates from livers and lungs of *Sdc1−/−* and wild type BALB/c mice under normal housing conditions were analyzed for activation of p38 (P-p38), and the effectors downstream of p38 signaling. Western blots were quantified to show relative p38α activation. **B**. Transcriptome analysis of liver mRNA showed some specific changes in *Sdc1−/−* livers. **C**. *Sdc1−/−* mice showed normal blood glucose levels until mice were cold-stressed (4°C, 90 mins) (n = 4). **D**. Body temperature was normal in *Sdc1−/−* mice until mice were cold-stressed (n = 7). **E**. Tissue lysates from peri-gonadal white adipose tissues (WAT) were analyzed for expression of UCP1, as a measure of cold activation and browning.

Interestingly, deficient intradermal fat expansion together with symptoms of chronic cold stress in *Sdc1−/−* mice were associated with systemic signaling changes in organs inside the body cavity. Thus, when the activation status of key metabolic and signaling hubs was assayed in lung and liver, p38α was consistently hyper-phosphorylated in *Sdc1−/−* mice (Fig. 3A). Assay of tissue lysates shows that the relative activation of p38α was higher in *Sdc1−/−* mice. Activation of p38α results in downstream activation of a key anti-cancer checkpoint, p53 [42], together with a number of regulators of cell cycle entry, including the cyclin-dependent kinase inhibitors, p16 (CDKN2A/p16^Ink4a^), p21 (CDKN1A/Waf1) and p27 (CDK1B/p27^Kip1^) [43]. Overall, analysis of mRNA expression in *Sdc1−/−* livers showed only moderate changes, including significant induction of 140 genes and decreased expression of 134 genes at a false discovery rate (FDR) of 10% (Table S1). Of these mRNAs, Elovl3 mRNA (Fatty acid elongase 3, for very long fatty acids) was increased by approximately 60-fold in *Sdc1−/−* livers (Fig. 3B), and has been previously associated with cold stress. Indeed, this enzyme was cloned because it was induced in brown adipose tissue after exposure to βadrenergic agonists or cold, and has been observed before in liver [36], [44]. Expression typically responds in order to compensate for changes in peroxisomal fatty acid oxidation [44]. There was also an 11-fold increase in expression of Gpr12 mRNA, a sphingosine-lipid activated G-protein coupled receptor which induces cAMP and downstream signaling pathways. Amounts of Fgf21 mRNA were strikingly variable, suggesting that this gene may be cyclically regulated or highly reactive to environment. Although expression trended higher in *Sdc1−/−* mice, the increases in this so-called “starvation hormone” were therefore not statistically significant [25]. Interestingly, there was also a 4-fold repression in a key transactivator of the Hippo pathway, Tead2. Inhibition of Hippo signaling is required for adipogenesis to proceed, and is mediated by sphingosine-associated lipid ligands of GPCR receptors [45]. Expression was significantly reduced for Defensin B1 (12-fold) and retinoic acid induced mRNAs (Raet1, 10-fold; Fig. 3B). We conclude that the changes observed were targeted and included mRNAs for proteins previously implicated in cold stress responses.

### Highly specific stress-response physiologies are altered in *Sdc1−/−* mice

*Sdc1−/−* mice showed other phenotypes consistent with their chronic cold stress. Thus, when exposed to 4°C cold for 90 minutes, *Sdc1−/−* mice showed decreased blood glucose (whereas normal mice did not) (Fig. 3C), presumably due to their low liver glycogen stores. Despite their lack of thermo-insulation, the body temperature of *Sdc1−/−* mice is normal at room temperature (*Sdc1−/−* mice, 37.3±0.17°C; BALB/c mice, 37.0±0.14°C). When exposed to an acute cold stressor (4°C for several hours), *Sdc1−/−* mice resisted the drop in body temperature that is typical for cold-naïve BALB/c mice (Fig. 3D). It is likely that this reflects a higher thermogenic capacity, which could be confirmed by measuring the thermogenic effect of a single norepinephrine injection. Furthermore, peri-gonadal WAT from *Sdc1−/−* mice showed seams of “browning” (observed histologically, Fig. S3) which was quantified by Western blotting of UCP1 protein (Fig. 3E) [8].

### Sdc1-associated phenotypes are rescued by relief of cold stress

**Figure 4:**
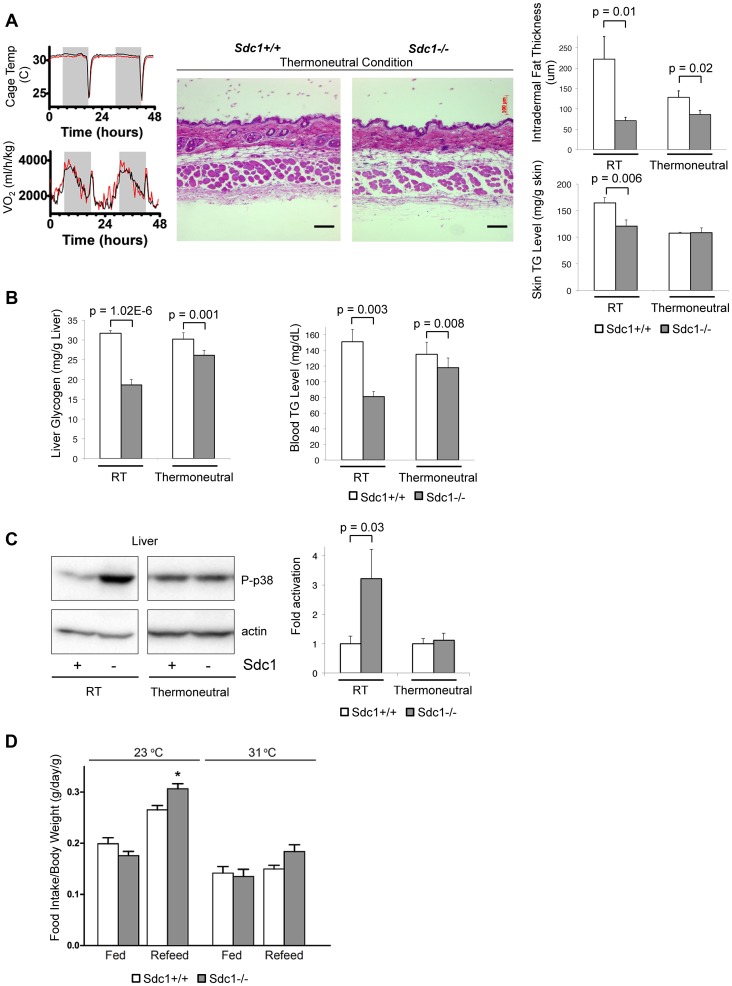
Thermoneutral housing rescues the *Sdc1−/−* phenotype. **A**. To illustrate the energy required to respond to a transition between 31°C (thermoneutral temperature) and 23°C, the oxygen uptake of mice in metabolic cages was measured (black, BALB/c; red, *Sdc1−/−*). Skin samples from mice housed at 31°C for 2 weeks were paraffin-embedded, sectioned, and H&E stained. Results showed that intradermal fat was thinner and almost equal for both BALB/c and *Sdc1−/−* mice (quantified at right hand side; n = 6). Skin triglycerides (TG) were also measured (triglyceride derives both from intradermal fat and from the epidermal layer itself, including the sebaceous glands). **B**. Liver glycogen and blood glucose levels are shown for mice housed at 31°C (and 23°C, RT), showing almost complete rescue. **C**. Tissue lysates from livers of mice housed at thermoneutrality showed that p38 activation in *Sdc1−/−* mice was equal to wild type BALB/c mice. **D**. Quantitation of relative food intake (fed, or fasted for 48 hours and re-fed) (n = 8 for each of *Sdc1−/−* and BALB/c wild type mice), to show that *Sdc1−/−* mice are hyperphagic during recovery from fasting.

Our hypothesis proposes that the metabolic phenotype of *Sdc1−/−* mice derives from a specific deficiency of intradermal fat, and the consequent cold stress. In order to test that proposal, we housed *Sdc1−/−* mice at thermoneutral temperatures. Under typical housing conditions, mice use considerable energy to maintain their body temperature, illustrated here as a spike of 50% increase in O_2_ consumption for either *Sdc1−/−* or wild type mice when the cage temperature was dropped from 31°C to 23°C for an hour (Fig. 4A). Thermoneutral conditions are defined as the temperature at which mice show basal metabolic rate; for mice this temperature range is 29–33°C [2], [12]. *Sdc1−/−* mice were therefore housed at 31°C for 2 weeks, to test whether Sdc1-associated phenotypes were reversed.

As expected, both control and *Sdc1−/−* mice showed thin layers of intradermal fat after acclimating to warm temperatures (Fig. 4A). In support of our hypothesis, liver glycogen stores and circulating blood triglycerides were restored to almost normal levels (Fig. 4B). Levels of p38α activation were low and equivalent for wild type and *Sdc1−/−* livers (Fig. 4C). *Sdc1−/−* mice showed minor/no statistical difference in energy expenditure (either at 23°C or 31°C, calculated per mouse or per g body weight, or using a multivariate regression analysis; Fig. S5B,C) [41], [46], [47], [48]. *Sdc1−/−* mice have a food intake approximately equal to wild type mice (per g body weight), and normal RER (Fig. S5), although they are relatively hyperphagic post-fasting (Fig. 4D). In sum, by housing *Sdc1−/−* mice in warmer temperatures, the intradermal fat layer thinned out to approximately match the intradermal fat layer of wild type mice. Under these conditions, all the systemic effects that we have described were rescued.

### Sdc1 is required for adipocyte differentiation

To investigate the molecular basis for this phenotype, we turned to previously published studies. Several groups have implicated the heparan sulfate associated with Sdc1 in lipid uptake from lipoprotein particles. Specifically, Esko and colleagues observed that Sdc1 was required for the uptake of VLDL particles by liver [19], [49]. Interestingly, those studies showed that circulating levels of triglycerides were higher in C57Bl6 *Sdc1−/−* mice; in contrast, we show that BALB/c *Sdc1−/−* mice have lower triglyceride levels. (Indeed, we confirmed the higher triglyceride levels in C57Bl6 *Sdc1−/−* mice: specifically, 74.7±7.9 mgs/dL; *Sdc1−/−*, 102.1±11.2 mgs/dL; p = 0.04). These strains are highly discrepant with respect to lipogenesis; indeed female BALB/c mice (like many strains) do not become obese on high fat feeding (data not shown, and [41], [50], [51]). Overall, the literature suggests that for cells not specialized for VLDL uptake, ie. cells other than white fat adipocytes, Sdc1 could be a key player in the capture of VLDL particles and the uptake of associated fats. Furthermore, Orlando and colleagues showed that over-expression of Sdc1 in fibroblasts was sufficient to confer the ability to take up the dye dissolved in the triglyceride in di-I-labeled VLDL particles, via an endocytic mechanism [52]. A prior study from Bernfield and colleagues showed that expression of Sdc1 was up-regulated early during adipogenesis in the 3T3-L1 cell line *in vitro*, and that Sdc1 stabilized lipoprotein lipase, important to mobilizing fatty acids from complex triglycerides [53]. We therefore hypothesized that knockdown of Sdc1 would inhibit the differentiation and/or the uptake of lipoprotein particles during adipocyte differentiation.

**Figure 5:**
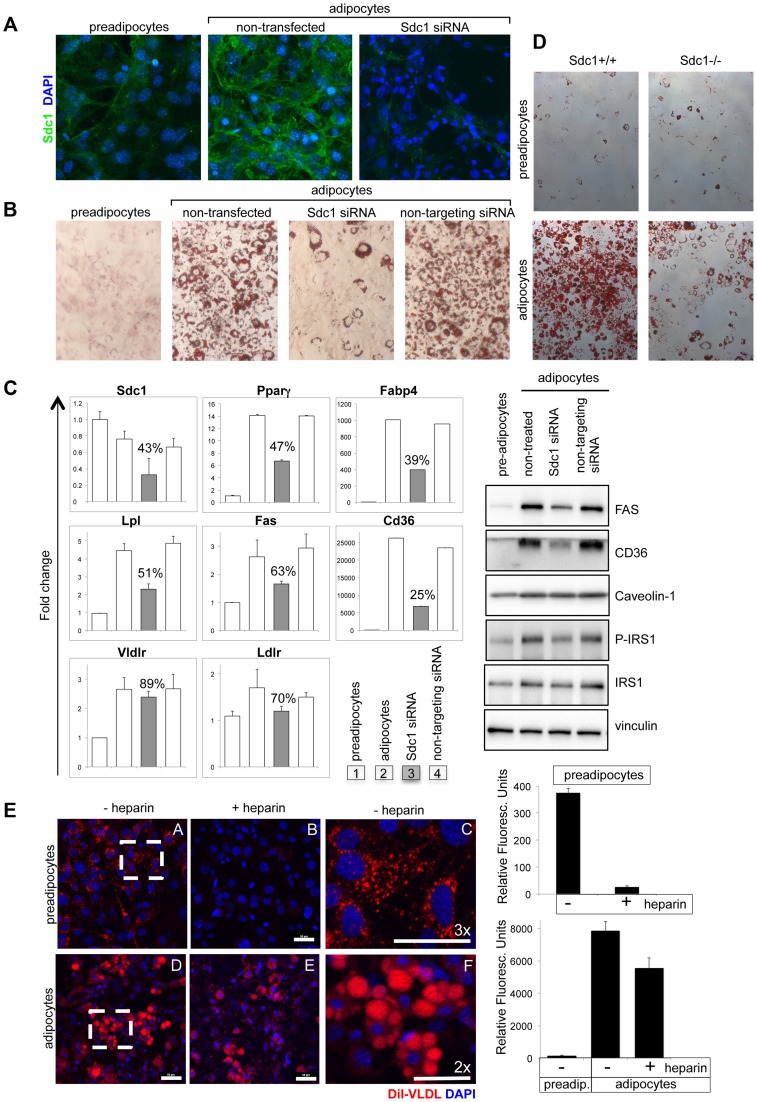
Loss of Sdc1 ablates adipocyte differentiation *in vitro*. **A**. 3T3-L1 cells (pre-adipocytes), 3T3-L1 cells 8 days after initiation of the differentiation protocol (adipocytes) and 3T3-L1 cells after knockdown of Sdc1 with siRNA were assayed for Sdc1 expression by immunofluorescent antibody staining. **B**. A similar series of cultured cells were stained with Oil Red-O, a dye that dissolves in lipid drops accumulating in differentiated adipocytes. **C**. Cell lysates were assayed for markers of differentiation by qPCR (PPARγ, peroxisome proliferator-activated receptor gamma; FABP4, fatty acid binding protein-4; LPL, lipoprotein lipase; FASN, fatty acid synthase; CD36, thrombospondin receptor) and by Western blotting (FasN, Cd36, activated phospho-IRS1). **D**. Ear mesenchymal stem cells (eMSCs) were isolated from *Sdc1−/−* and BALB/c mice, transferred to culture, and induced to differentiate. Differentiation was visualized by Oil Red-O staining. Corresponding nuclear stains (Fig. S6C) illustrate that the cell densities are approximately similar. **E**. To evaluate the impact of a heparan sulfate mimetic, heparin, on the ability of 3T3-L1 preadipocytes (A,B,C) and adipocytes (D,E,F) to take up VLDL, cells were incubated in presence (B,E) or absence (A,C,D,F) of 200 µM heparin with di-I labeled VLDL for 3 hours at 37°C. Fields from A and D were magnified (C and F respectively) to show the size and morphology of the red-stained vesicles. Uptake was quantified (right hand side panel). Lower concentrations of heparin showed similar effects (50 µM; data not shown).

We determined the effect of Sdc1 knock-down on the differentiation of 3T3-L1 cells, a culture model of adipocyte differentiation. We confirmed that Sdc1 protein was expressed and induced during adipocyte differentiation (Fig. 5A), as has been reported previously [53]. Knock-down of Sdc1 (using siRNA) profoundly reduced the accumulation of intracytoplasmic lipid inclusions that is characteristic of adipocyte differentiation (revealed by Oil Red O staining; Fig. 5B).

Mechanistically, we hypothesized that lack of fat accumulation by adipocytes could be the result of either reduced uptake of serum-associated VLDL, or the inhibition of differentiation of 3T3-L1 cells. To distinguish between these options, differentiation-associated markers were assayed in Sdc1 knock-down 3T3-L1 cultures: expression of reporter mRNA species such as PPARγ (peroxisome proliferator-activated receptor gamma, the hub of adipogenic differentiation-associated transcription), FABP4 (fatty acid binding protein-4), Lpl (lipoprotein lipase), FASN (fatty acid synthase) and CD36 (thrombospondin receptor) [54], [55], [56], together with several adipocyte-associated proteins (FasN, Cd36, activated phospho-IRS1). These were all reduced in parallel to Sdc1 knockdown (Fig. 5C).

**Figure 6:**
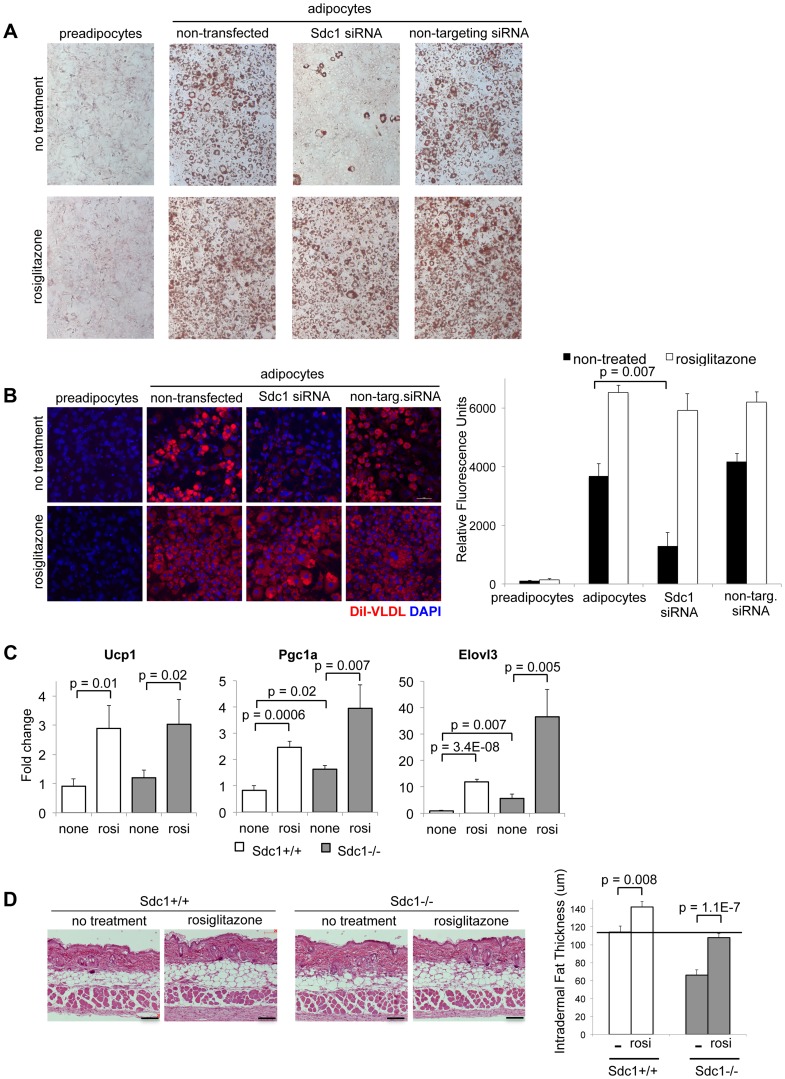
Adipocyte differentiation can be rescued *in vitro* and *in vivo* by the PPARγ agonist, rosiglitazone. **A**. 3T3-L1 cells, treated as indicated, were stained for Oil Red O to assess their differentiation. Rosiglitazone (2 µM) was added with the differentiation medium for 2 days. **B**. For similar cultures, VLDL uptake was measured. **C**. Rosiglitazone (0.015% diet for 5 days) was administered to *Sdc1−/−* and wild type mice, and reporters of pPARγ activity (mRNAs for Ucp1, Pgc1α and Elovl3) were measured by qPCR in mRNA extracts of white adipose tissue. **D**. Skins from rosiglitazone- and control-diet fed mice were paraffin embedded and sectioned to determine the thickness of intradermal fat (n = 8).

These experiments were repeated using ear mesenchymal stem cells (eMSCs) from BALB/c and *Sdc1−/−* mice; eMSCs are a primary cell culture model useful for the interrogation of genetically modified mouse strains [57], [58], [59]. Immunohistological sections of ear show that this is a rich source of intradermal differentiated adipocytes along with undifferentiated cells (Fig. S6D). These cells show a similar pattern of Sdc1 expression during differentiation as 3T3-L1 cells (Fig. S6A). This model of adipocyte differentiation also required Sdc1 for differentiation, shown by loss of Oil Red O staining (Fig. 5D; matching nuclear stains are shown in Fig. S6C). There was reduced expression of differentiation-associated mRNAs by *Sdc1−/−* eMSCs (Fig. S6B). This was reproduced in *Sdc1−/−* eMSCs of three different strains (BALB/c (Fig. 5D and S6) and for C57BL6 and FVB mice, Fig. 6C). Overall, we conclude that loss of function of Sdc1 inhibits the differentiation of pre-adipocytes.

### Heparan sulfate is important for VLDL-tG uptake in pre-adipocytes

Since Sdc1 (more specifically, the heparan sulfate associated with Sdc1) has previously been shown to bind VLDL and mediate the uptake of triglycerides (tG), we evaluated the effect of the competitive inhibitor of heparan sulfate, heparin, on VLDL-tG internalization in this cell model. We found that the uptake of di-I labeled VLDL-tG was dramatically inhibited in pre-adipocytes (90%) in the presence of heparin (Fig. 5E). In contrast, VLDL uptake was almost heparin-independent in adipocytes. Since adipocytes express several specific lipoprotein receptors (for example VLDLR and LDLR; mRNA expression is shown in Fig. 5C), it is likely that heparan sulfate proteoglycans are redundant and functionally insignificant.

### Sdc1 acts upstream of PPARγ, as a component of the trigger mechanism for adipocyte differentiation

This outcome suggested that Sdc1 is part of the trigger mechanism for adipocyte differentiation. Since activation of PPARγ by synthetic ligands reduces the need for an upstream adipogenic trigger, we treated 3T3-L1 cells with the PPARγ agonist, rosiglitazone (Rosi). Rosiglitazone induced robust adipocyte differentiation, regardless of Sdc1 (Fig. 6A), suggesting that the activity of Sdc1 is required upstream of PPARγ activation. Reduced Sdc1 activity had no effect on VLDL uptake by adipocytes induced by rosiglitazone administration (Fig. 6B), confirming that Sdc1 is not an important component of exogenous lipoprotein uptake in differentiated adipocytes.

Since a PPARγ agonist can rescue the effect of loss of Sdc1 *in vitro*, we tested whether this rescue would also work *in vivo*. There are no specific reports in the literature that describe the modulation of intradermal fat in response to any drug, despite a wealth of data that describe changes of metabolism induced specifically by various PPAR agonists [60]. Note that intradermal fat is not the same as subcutaneous fat, though definitions are sometimes not explicit. We treated mice with rosiglitazone for 5 days, a treatment that induced classic PPARγ- dependent changes in white adipose tissues, including the induction of mRNAs for proteins important to uncoupling/browning of white adipose tissue (UCP1, PGC1α and Elovl3; Fig. 6C). We found that rosiglitazone administration increased the amount of intradermal fat in wild-type mice; indeed, detailed examination of the histology of the skins of mice similarly treated by Varga and colleagues showed similar changes [61]. More importantly for this study, this treatment substantially rescued the intradermal fat layer of *Sdc1−/−* mice (Fig. 6D). This supports our proposal that the absence of Sdc1 generates a cell-autonomous loss of differentiation in intradermal pre-adipocytes that results in a deficient thermal “blanket” and chronic, unalleviated cold stress.

## Discussion

Many responses to cold have been documented, and these differ according to the severity of the cold stress, and whether the cold stress is acute or chronic. Studies of mice illustrate the redundancy of mechanisms, since different strains emphasize different strategies [8]. They include increased length of fur, altered vascularization, decreased metabolic and activity rates, and lower body temperatures; these are accompanied by behavioral responses such as nest building, neural adaptation and shivering [37], [62], [63], [64]. Interestingly, we found that *Sdc1−/−* mice show substantial defects in a dynamic layer of adipocytes localized under the epidermis in skin, called intradermal fat. Its thickness is regulated by ambient temperature, notably across the 31–20°C temperature range from thermoneutrality to mild cold stress. Skin provides the only barrier that modulates heat loss from body temperature (37°C) to the environment (RT/22°C). We calculated the theoretical thermal conductivity of mouse skins containing a thin 40 µM layer of adipose tissue (observed in *Sdc1−/−* mice at 22°C, and all mice housed at thermoneutrality), and the thicker 200 µM layer observed at 22°C in BALB/c mice (Fig. S7). There is a 1.8-fold increase in heat loss through a 40 µM intradermal fat layer compared to skin containing 200 µM (all other factors being equal). This layer of insulating adipose tissue could therefore have a surprisingly key role for physiology; furthermore, the mechanism that regulates this highly dynamic tissue is likely to be an important determinant of metabolism.

We have shown that *Sdc1−/−* mice show symptoms of abnormal cold stress at normal housing temperatures. Thus the thermogenic response of BAT is activated, there is evidence of browning in WAT, and mice are susceptible to fasting-induced torpor. They show chronic cold stress-associated phenotypes such as glycogen depletion and depleted circulating triglycerides [22]. Altered demands on liver function are reflected by the induction of the biosynthetic enzyme, Elovl3, also a marker of cold stress [36], [44] and Pgc1α. These mice show abnormal responses to acute cold stressors; this is typical of chronically cold-adapted mice, or animals pre-exposed to cold [31], [65].

Room temperature housing is already known to be an important determinant of specific physiologies. Thus the obese phenotype of UCP1 mice was not apparent until mice were moved from 20°C to 29°C housing [12], [33]. Macrophages in brown and white adipose tissues showed significant alternative activation in mice housed at 22°C (or acutely challenged at 4°C) compared to those housed at 30°C [5]. Indeed, absent macrophages impaired metabolic adaptations to the cold. Antigen-specific (tumor-directed) CD8-positive T cells were activated in mice housed at 31°C compared to 22°C [11], leading to much-reduced tumor growth in warm temperatures. These two immunosuppressive cell types therefore show opposite trends for activation in cool and warm temperature housing, so the net result of ambient temperature for any given physiology may be difficult to predict *a priori*. Both cells types secrete, and are governed by, systemic cytokines.

There is another example of a skin-associated phenotype that is known to generate a cold stress, by a contrasting mechanism. Thus *Scd1*(stearoyl-CoA desaturase)-deficient mice show depleted Δ9 monounsaturated 16∶1 and 18∶1 fatty acids in skin sebocytes. This results in loss of skin barrier homeostasis and acute cold sensitivity [66], . There are similarities and differences between *Scd1−/−* mice and *Sdc1−/−* mice; they both show hyperactive BAT, and widespread up-regulation of thermogenic response genes (UCP proteins and PGC1α in muscle, BAT and WAT). However unlike *Scd1−/−* mice, *Sdc1−/−* mice do not show a highly elevated metabolism rate, acute cold sensitivity, or exacerbation of metabolic hyperactivity and dehydration at thermoneutral temperatures, reflecting the difference in underlying mechanisms.

Note that at this point we cannot exclude the possibility that there is an altered demand for heat generation due to the relatively higher surface area/volume ratio of the smaller (13%) Sdc1 mice. Indeed, this factor applies in general to metabolic studies of mouse strains that vary in size [41], [46], [47], and to genetically modified mice that are smaller than their control counterparts (for example, Fgf21 transgenic mice (40–50% smaller) [68], *Igf1−/−* (69%) [69] and *ApoE*−/− (22%) [70]). However, results from Sdc1 knockdown (or knock out) in pre-adipocytes, suggest that Sdc1 is required to trigger adipocyte differentiation, leading us to hypothesize that the systemic effect of absent Sdc1 may reflect a cell-autonomous function in intradermal adipocytes.

Different types of adipocytes are programmed to respond to cold stress is different ways. Thus, white adipose tissues are induced to release triglyceride stores, and induce a browning/beige response. Brown adipose tissues are induced to take up lipids, mobilize their own, and induce thermogenesis via uncoupling of mitochondria utilizing β−oxidation of fatty acids [6], [9], [71], [72], [73]. Our observations suggest that intradermal adipocytes have a distinct response; they accumulate lipid in response to cold stressors, and the intradermal fat expands by 4-fold. In common with other types of adipocytes, these intradermal adipocytes are depleted in response to a PPARα agonist (WY14643; data not shown).

We have shown that loss of Sdc1 dramatically inhibits differentiation of adipocytes *in vitro*. Intradermal fat is highly kinetic compared to other adipose reserves, expanding and collapsing every month, and adjusting in response to ambient temperature. We propose that this underlies the highly specific nature of the lesion produced by absent Sdc1 (the size of WAT deposits are not affected in *Sdc1−/−* mice). We have shown that Sdc1 is important for VLDL uptake in pre-adipocytes, and suggest that this may be important to its role in differentiation. The PPARγ agonist, rosiglitazone, can rescue the effects of Sdc1 deficiency, and we conclude that Sdc1 is likely to be a component of the sensory trigger for intradermal fat differentiation.

**Figure 7:**
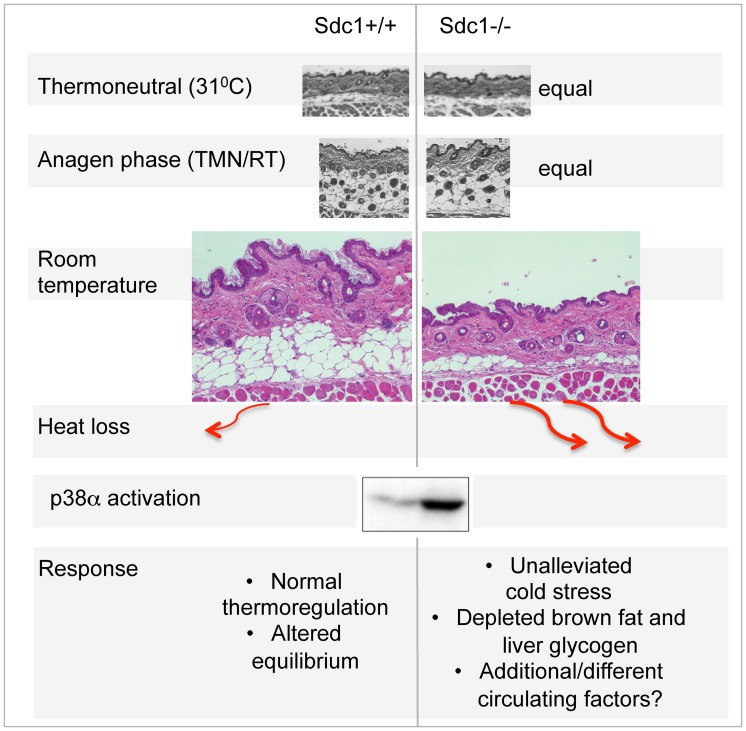
Summary scheme of the effects of deficient intradermal fat in *Sdc1−/−* mice. The thickness of intradermal fat in non-anagen phases is set by ambient temperature, and is 80% depleted in *Sdc1−/−* mice housed at room temperatures. During anagen phase (when intradermal fat expands in response to local cues), the thickness of *Sdc1−/−* intradermal fat is high and normal; in warm temperatures, the intradermal fat of *Sdc1−/−* mice is thin and normal. Heat loss from skin containing 40 µM intradermal fat is calculated to be at least 2-fold higher than skin with 200 µM of intradermal fat. This heat loss leads to systemic p38α activation throughout intra-abdominal tissues, and a condition of “unalleviated cold stress” in *Sdc1−/−* mice.

No other studies have monitored the response of intradermal fat over this temperature range. We hypothesize that mice with a sufficient insulating response are able to alleviate mild cold stress, albeit altering their metabolic equilibrium, probably with chronic changes of systemic cytokines. In contrast, though the body temperature is maintained in *Sdc1−/−* mice exposed to cool housing temperatures, the intradermal fat layer of *Sdc1−/−* mice does not expand sufficiently to alleviate this stressor. This results in “unalleviated” cold stress, notably associated with systemic p38α activation (Fig. 7). *Sdc1−/−* intradermal fat shows normal depletion in warm temperature housing, and normal expansion during the hair cycle. (Adult mouse skin is in anagen stage almost half the time, and the hair cycles are asynchronous, in patches throughout the mouse pelt [74], [75]). The implication is that Sdc1 is involved only in the cold-sensitive trigger for adipocyte differentiation. The dynamic process of intradermal fat accumulation and involution, together with the effect of Sdc1, is illustrated in Fig. 7.

MAPK14/p38 is an essential mediator of the cold stress response for brown adipose tissues, inducing the expression of the uncoupler protein, UCP1, in response to β3-adrenergic stimulation of PKA [26], [76]. This is one of two types of MAPK pathway dedicated to “stress” responses; it is also activated in response to stressors that are osmotic, oxidative, or immune in origin for other cell types, and modulates outcomes for a great variety of signaling pathways [43], [77]. Specifically, p38α activation leads to activation of anti-cancer checkpoints p53 and CDK inhibitors [78], and is known to control susceptibility to tumor development and metastasis [43], [79]. Typically, by inducing thermogenesis in BAT, β3-agonists relieve cold stress, and suppress further responses. However, in *Sdc1−/−* mice, the effects of cold stress are inadequately buffered, and the activation of p38α becomes widespread, throughout the intraperitoneal organs of *Sdc1−/−* mice. We predict that this could have the side effect of enhanced protection against the development of pathologies typically held in check by p38α.

In conclusion, we suggest that without Sdc1, the ability of intradermal adipocytes to respond to environmental temperature cues is impaired. This leads to an inadequate thermogenic response and a disproportionate cold stress at temperatures below thermoneutral conditions. Humans and mice have similar strategies for thermoregulation, which is a conclusion emphasized by the relatively recent discovery of human BAT [80], [81]. There may be opportunities to inhibit the process of intradermal fat differentiation (specifically inhibiting the Sdc1-mediated function) to engage the systemic hyper-activation of stress checkpoints in both mice and men.

## Materials and Methods

### Reagents and antibodies

Heparin sodium salt, fatty acid free bovine serum albumin, dexamethasone, 3-isobutyl-1-methylxanthine (IBMX) and insulin were all from Sigma-Aldrich (St Louis, MO). DiI-labeled Very Low Density Lipoprotein (VLDL) was obtained from Kalen Biomedical (Montgomery, MD). Paraformaldehyde was from Electron Microscopy Sciences (Hatfield, PA). Rosiglitazone was from Cayman Chemicals (Ann Arbor, MI). Oil red O was obtained from Amresco (Solon, OH). Antibodies used for immunoblot or staining were anti-p38, P-p38, p38α, p38γ, P-p53, p27, FAS, caveolin-1 (Cell Signaling Technology, Danvers, MA), p16 (Santa Cruz Biotechnology, Santa Cruz, CA), β-actin (Sigma-Aldrich), p21, p-IRS1 and anti-mouse IgG-Alexa 488 (Invitrogen, Carlsbad, CA), CD36 and UCP1 (Abcam, Cambridge, MA), vinculin (Chemicon, Billerica, MA), donkey-anti-mouse IgG-HRP and normal goat serum (Jackson Immuno Research Laboratories, West Grove, PA), and goat-anti-rabbit IgG-HRP (Molecular Probe, Carlsbad, CA). S1ED anti-mouse syndecan1 antibody was a kind gift from Dr. Alan C. Rapraeger [82].

### Mice, diet and metabolic cage measurements

The generation of *Sdc1−/−* mice has been described previously (McDermott et al 2007). Note that all the assays describe BALB/c female mice, except where indicated. Mice were housed at room temperature (20–23°C) unless otherwise specified. For isothermal housing, mice were individually caged, housed at 31°C (±1°C) for 2 weeks in a controlled environment facility, and monitored daily. For cold tolerance testing, mice were individually housed in a cold room (cage temperature: 4∼5°C). A Thermalert TH-5 monitoring thermometer with a RET-3 mouse rectal probe was used to measure body temperature. Mice were maintained on a 12 h light and dark cycle with free access to water and chow diet (LabDiet# 5020, St Louis, MO or Teklad#8604, Harlan Laboratories, Madison WI). To administer a PPARγ agonist to mice *in vivo*, diet was formulated with Rosiglitazone (0.0015%) by Harlan Laboratories (Madison, WI). Serum was prepared for analysis and kept at −20°C until needed. Samples for skin sections were paraformaldehyde-fixed (4%) overnight and then paraffin-embedded for evaluation. For individual assay of multiple metabolic parameters (O_2_, CO_2_, food and water consumption), mice were transferred to a LabMaster modular animal monitoring system from TSE Systems (Chesterfield, MO), acclimated for 1 week prior to measurements, and phenotyped as described [47], [48], [83].

### DXA analysis

BALB/cJ stock and *Sdc1−/−* BALB/cJ mice 7 to 12 weeks of age were anesthetized (3% isoflurane) and scanned using a Lunar PIXImus densitometer (GE/Lunar Corp, Madison, WI). Calibration of the instrument, animal placement, and scan analysis were conducted as suggested by the manufacturer. One investigator did all DXA determinations. All data presented for body composition exclude the head, by placing an exclusion region of interest (ROI) over the head.

### Physiological glucose, glycogen and triglyceride measurements

Blood glucose level was measured from serum using QuantiChrom glucose assay kit (BioAssay Systems, Hayward, CA) that utilizes an improved o-toluidine method. In some experiments, blood glucose level was measured directly from the mice after bleeding at the tail tip with OneTouch Ultra 2 Blood Glucose Monitoring System (LifeScan, Milpitas, CA). Liver glycogen amount was measured as follows: liver tissues were homogenized in PBS with a Polytron PT2100 (Kinematica, Lucerne, Switzerland). Liver glycogen amount was measured using a colorimetric enzymatic procedure with minor modifications [84]. Briefly, liver tissues were homogenized with a Polytron PT2100 (Kinematica, Lucerne, Switzerland) and amyloglucosidase (Roche Diagnostics, Indianapolis, IN) was added for 10 mins at 55°C to break down glycogen to glucose; aliquots were added to a reaction mixture containing glucose oxidase (MP Biomedicals, (Solon, OH); mixture includes peroxidase from MP BioMedicals, N,N-dimethylanaline and 4-aminoantipyrine from Acros Organics (Pittsburgh, PA)) for 30 mins at 37°C, and absorbance was measured at 550 nm. Triglycerides in serum were measured using the EnzyChrom triglyceride assay kit from BioAssay Systems (cat#ETGA200). Tissues were freshly ground frozen, mixed with extraction buffer (isopropanol∶Triton-X100∶water = 5∶2∶2 (v/v)) and homogenized. After a brief centrifugation at 14000 g for 5 min, 4°C, the supernatant containing extracted triglyceride was collected and processed as described by the manufacturer.

### Ethics statement

This study was performed in strict accordance with the recommendations in the Guide for the Care and Use of Laboratory Animals of the National Institutes of Health. Experimental protocols were approved by the University of Wisconsin School of Medicine and Public Health Animal Care and Use Committee. The number of mice used to perform this study was minimized, and every effort was made to reduce the chance of pain or suffering.

### Western blot analysis

Tissues were dissected and stored in liquid nitrogen until analysis. Frozen tissue was ground into powder and homogenized in RIPA lysis buffer (20 mM Tris-HCl, pH 7.5, 150 mM NaCl, 1 mM EDTA, 1 mM EGTA, 1% NP-40, 1% sodium deoxycholate) supplemented with protease and phosphatase inhibitors (Thermo Scientific, Rockford, IL). Protein concentration was determined using the BCA protein assay. Lysates were analyzed by SDS-PAGE, followed by transfer to PVDF membranes and assay using the antibodies described.

### Quantitative RT-PCR analysis and microarray processing

Total RNA was isolated from cells and most tissues using the RNeasy Mini Kit (Qiagen, Valencia, CA); for fatty tissues the RNeasy Lipid Mini Kit (Qiagen) was used instead. Array processing, and cDNA synthesis for qPCR assay was done according to the Supplemental Experimental Procedures.

### Cell culture, adipocyte differentiation, knock-down of Sdc1, immunofluorescent staining and ear mesenchymal stem cell preparation

Mouse 3T3-L1 preadipocytes were from the American Tissue Culture Collection (ATCC). Cells were maintained in Dulbecco's modified Eagle's medium supplemented with 4.5 g/L of glucose (Life Technologies), 10% fetal bovine serum and 100-U/ml penicillin and streptomycin. Cells were differentiated into adipocytes as described by others (Wilsie et al., 2005); briefly, confluent 3T3-L1 preadipocytes were induced to differentiate using MDI medium (100 µg/ml 3-isobutyl-1-methylxanthine, 100 ng/ml dexamethasone and 1 µg/ml insulin) for 4 days, followed by 1 µg/ml insulin for an additional 4 days. For some experiments, the PPARγ agonist, rosiglitazone (2 µM) was added to MDI media for 2 days, and cultures were re-fed every 2 days. To knock-down Sdc1, 3T3L1 preadipocytes were transfected with Sdc1 siRNA (Dharmacon ThermoScientific, Rockford, IL) using Lipofectamine RNAiMAX Transfection Reagent (Life Technologies) according to the manufacturer's instructions. Briefly, cells were seeded at 90% confluency in either 4 well chambers or 24 well plates. On the next day, cells were transfected with 15 pmol of anti- Sdc1 or non-targeting siRNA. To provide a gross assay of lipid accumulation, the formation of oil droplets in cells was analyzed using Oil Red O staining. Cells were fixed for 60 mins at 23°C with 3% paraformaldehyde, and stained with filtered 0.21% Oil Red O solution for 10 min, followed by four washes with PBS. Cells were photographed, and accumulation was quantified by dissolving the dye in isopropanol and measuring the optical density at 510 nm. Skin samples were taken from the same anatomical site (either belly or back, as indicated), paraformaldehyde-fixed (4%) overnight and oriented during paraffin-embedding. Tissues sections were deparaffinized, re-hydrated and stained with H&E. Cultured cells were fixed with 3% paraformaldehyde for 20 min. After blocking with 5% normal goat serum for 30 mins at room temperature, cells were incubated overnight with a rabbit polyclonal anti mouse-Sdc1 “S1ED” antibody (gift from Dr. Alan Rapraeger), and the anti-mouse IgG-Alexa 488 secondary antibody for 1 h at room temperature. Ear mesenchymal stem cells were prepared according to the methods described by Rim et al (2005) and Mori and MacDougald [57], [58], and differentiation was induced as for 3T3-L1 cells, with the following modifications: eMSCs were incubated in an MDI differentiation medium (containing 0.5 mM IBMX, 1 µM dexamethasone and 1.7 µM insulin) for 5 days, followed by DMEM/F12 culture medium with 10 nM insulin for 3 more days.

### DiI-labelled VLDL uptake assay

VLDL uptake by preadipocytes and adipocytes was assayed as previously described (Wilsie et al., 2005) with some minor modifications. 3T3-L1 preadipocytes were seeded, or matured adipocytes differentiated, on 4 well glass chamber dishes. Cells were incubated with DiI-VLDL (4 µg/ml) diluted into DMEM with 1% fatty acid free bovine serum albumin (FAF/BSA) in the presence or absence of either heparin (200 µg/ml). After 3 h at 37°C, cells were fixed with 3% paraformaldehyde for 20 min, and mounted in ProLong Gold Antifade Reagent with DAPI (Life Technologies). Cells were visualized on a confocal microscope (BioRad MRC1024), and fluorescence intensity was quantified by Image J software.

### Statistical analysis

Data are expressed as mean +/− standard error of the mean and statistical analysis was performed with unpaired one-tailed t tests using Microsoft Excel software. The results with calculated P values less than 0.05 are considered statistically significant.

## Supporting Information

Figure S1 Immunohistochemical analysis of mitotic index and Sdc1 expression in skin. **A**. Skin sections from *Sdc1−/−* and Balb/c mice were stained with Ki67 (mitotic index marker) and anti- Sdc1 antibody. Examination of these sections showed that the mitotic index of *Sdc1−/−* follicles was normal, despite the induction of Sdc1 expression in epithelial cells during anagen. **B**. Pups of both wild type and *Sdc1−/−* genotypes were photographed daily to record the first (synchronous) hair cycle, which did not vary between genotypes.
(TIF)

Figure S2 Evaluation of skins from *Sdc1−/−* and BALB/c mice. To interrogate further the collagen distribution and ECM structure, skins were subjected to Picro Sirius Red staining. Samples showed a range of morphologies in the sub-dermal muscle layer, depending on the orientiation of myofibril bundles. Various examples are shown, including one of anagen-stage from each genotype.
(TIF)

Figure S3 Comparison of white adipose tissues from *Sdc1−/−* and BALB/c mice. **A**. Cross sections of white adipose tissues (gonadal/peri-uterine) showed that the size of adipocytes was approximately similar for *Sdc1−/−* and BALB/c mice (and weights of WAT were not different). However, there was evidence of browning, revealed as seams of lipid-depleted adipocytes (confirmed by the more quantitative analysis by Western blotting; Fig. 3F). **B**. mRNA was extracted from white adipose tissue from *Sdc1−/−* and BALB/c mice and the relative expression of differentiation-associated mRNAs was assayed (CD36, FABP4, FASN, LPL, PPARγ). Expression of markers connected with WAT function were not significantly affected in *Sdc1−/−* mice.
(TIF)

Figure S4 Synergy of hair follicle invagination and expansion of skin-associated tissue layers. Data redrawn from Hansen et al (1984), showing hair regrowth for SAS/4 albino mice, depilated at 11 weeks of age.
(TIF)

Figure S5 Comparison of metabolic parameters in Sdc1−/− mice housed at various temperatures. **A**. Timecourse plots from the experiments shown in Figs. 1 and 4 are shown, to compare the RER of BALB/c (black lines; n = 4) and *Sdc1−/−* mice (red lines; n = 4), and illustrate the VO_2_ and energy expenditure of mice without body weight corrections. **B**. Quantitation (energy expenditure, averaged over 48 hours and expressed either per mouse, or corrected for body weights) of metabolic rates in mice housed at various temperatures (20°C, 23°C and 31°C), supplemental to Figs. 1C, D and 4D. **C**. Multivariate regression analysis of the impact of genotype on energy expenditure. To test whether extra energy expenditure (**EE**) was observed in *Sdc1−/−* mice (absent adequate biological insulation), we performed a multivariate regression analysis, using the online tool available at the National Mouse Metabolic Phenotyping Centers (http://www.mmpc.org/shared/regression.aspx). The effect of size and genotype on energy expenditure was compared (n = 8 for each cohort). There was a significant effect of body weight (**BW**) on EE (as expected given that *Sdc1−/−* mice are 13% smaller; p = 0.005), but no effect of genotype (p = 0.45).
(TIF)

Figure S6 Differentiation of adipocytes in eMSC cultures from *Sdc1−/−* mice. **A**. Cultured eMSCs (preadipocytes) were fixed, and immunostained for Sdc1, to show that Sdc1 was expressed in both stages of differentiation, and no Sdc1 was expressed in eMSCs from *Sdc1−/−* mice. **B**. Various mRNAs were assayed by qPCR to assess the relative differentiation of *Sdc1−/−* eMSCs (for comparison with Fig. 5). **C**. Nuclear stains (Hoecsht) of BALB/c *Sdc1−/−* and wild type eMSC cultures (to match with Fig. 5D), and from Oil Red-O stained eMSC cultures from C57Bl6 and FVB *Sdc1−/−* and wild type mice, before (pre-adipocytes, top row) and after induction of differentiation (adipocytes, bottom row). **D**. The source of eMSCs, the mouse outer ear, is illustrated in an H&E-stained section, showing the central cartilage band and layers of adipocytes on both sides.
(TIF)

Figure S7 Calculation of thermal conductivity of skins with thin and thicker intradermal fat. The thickness of various layers of skin (dermis, epidermis and intradermal fat; see also Fig. S4), together with their published thermal conductivities, were used to calculate the thermal resistance of 200 µM (top dataset) and 40 µM (bottom dataset) of skin. The result suggests that decreased fat layer could cause a 1.8× increase in heat flow/unit area.
(TIF)

Table S1 Array data from *Sdc1−/−* liver mRNA. Liver mRNA samples (3 independent samples for *Sdc1−/−* and BALBc mice) were subjected to array analysis, and data normalized as described in Experimental Procedures. Significantly increased and decreased mRNA species in *Sdc1−/−* livers are listed as genes UP and DOWN (significance assessed as p<0.05).
(XLSX)

Text S1 Details are provided for immunofluorescence staining, microarray processing and qPCR methods.
(DOCX)
