# Supplementary figures and images for: Syndecan-1 Is Required to Maintain Intradermal Fat and Prevent Cold Stress
Source: PLoS Genet. 2014 Aug 7;10(8):e1004514. doi: 10.1371/journal.pgen.1004514 (PMC4125098; doi:10.1371/journal.pgen.1004514)

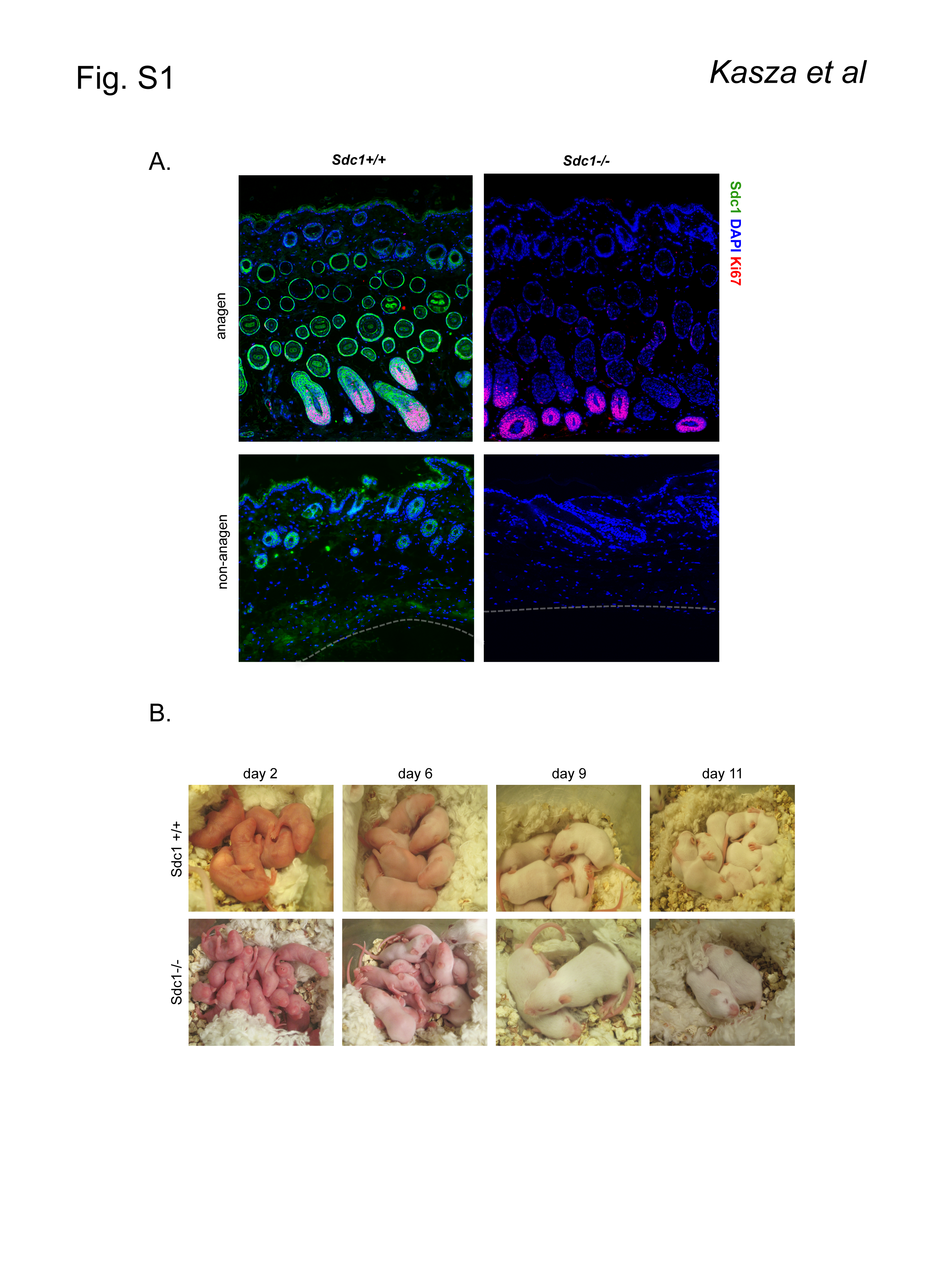

Supplement: Figure S1 — Immunohistochemical analysis of mitotic index and Sdc1 expression in skin. A. Skin sections from Sdc1−/− and Balb/c mice were stained with Ki67 (mitotic index marker) and anti- Sdc1 antibody. Examination of these sections showed that the mitotic index of Sdc1−/− follicles was normal, despite the induction of Sdc1 expression in epithelial cells during anagen. B. Pups of both wild type and Sdc1−/− genotypes were photographed daily to record the first (synchronous) hair cycle, which did not vary between genotypes. (TIF) [file pgen.1004514.s001.tif]

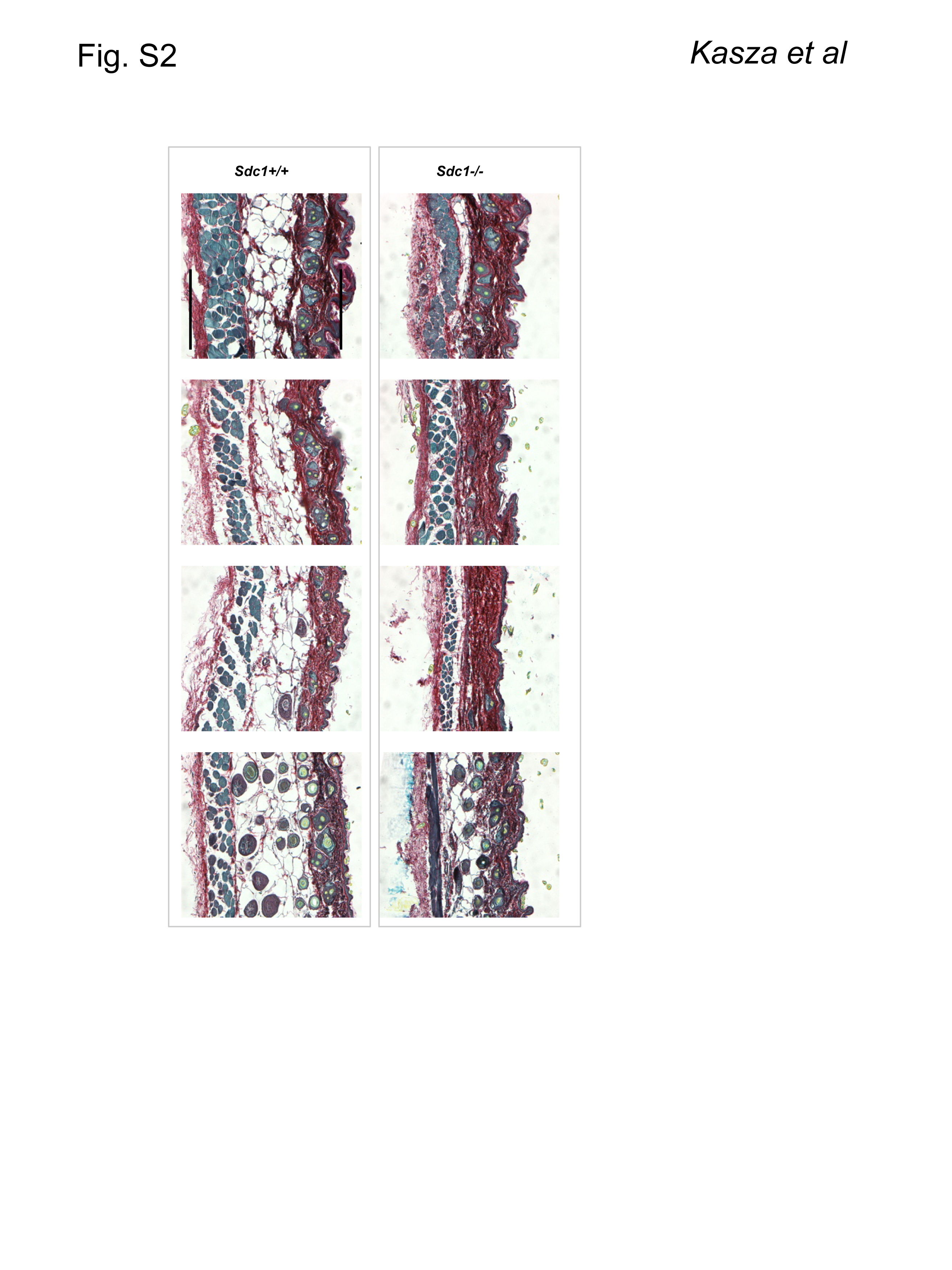

Supplement: Figure S2 — Evaluation of skins from Sdc1−/− and BALB/c mice. To interrogate further the collagen distribution and ECM structure, skins were subjected to Picro Sirius Red staining. Samples showed a range of morphologies in the sub-dermal muscle layer, depending on the orientiation of myofibril bundles. Various examples are shown, including one of anagen-stage from each genotype. (TIF) [file pgen.1004514.s002.tif]

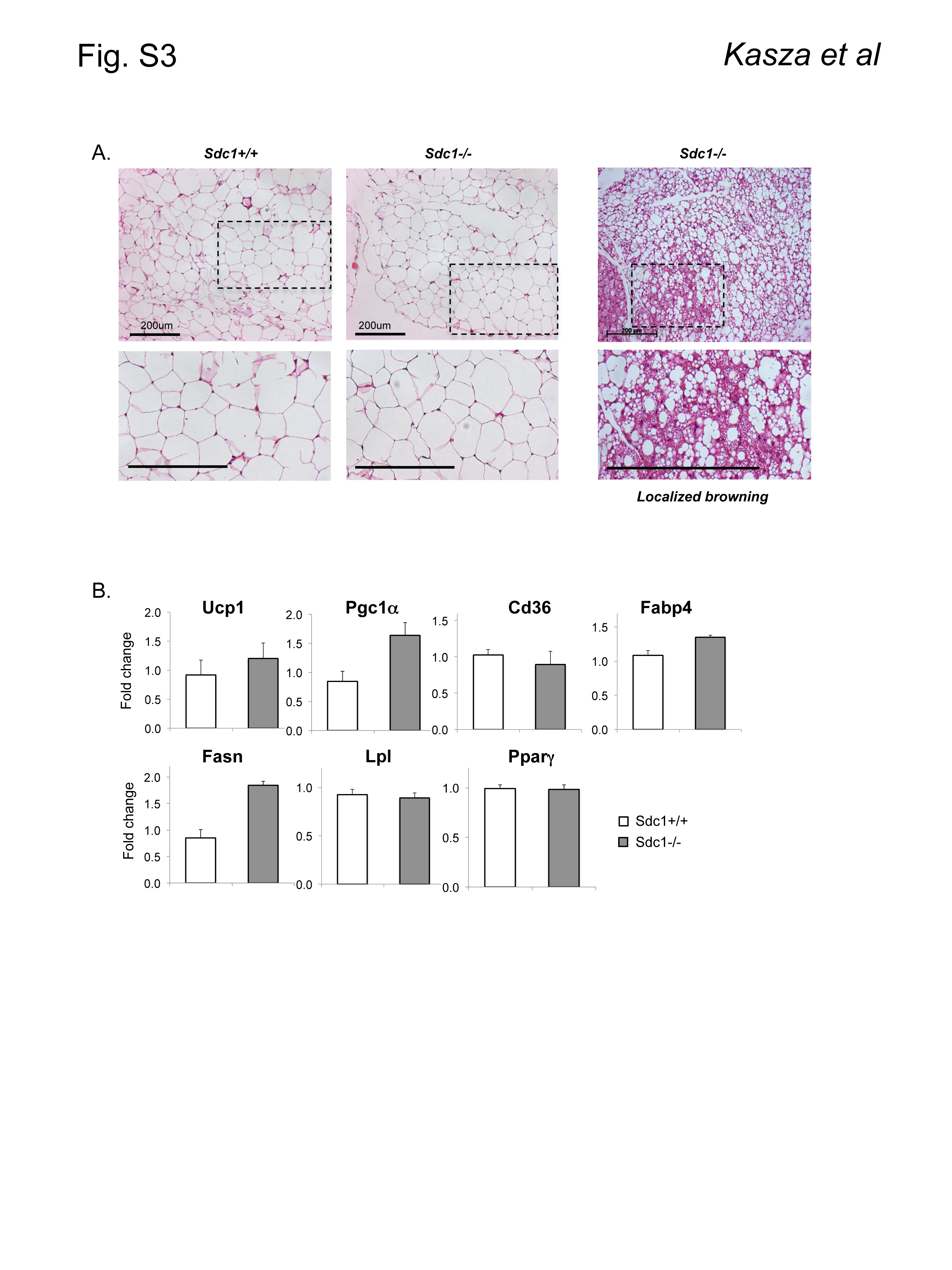

Supplement: Figure S3 — Comparison of white adipose tissues from Sdc1−/− and BALB/c mice. A. Cross sections of white adipose tissues (gonadal/peri-uterine) showed that the size of adipocytes was approximately similar for Sdc1−/− and BALB/c mice (and weights of WAT were not different). However, there was evidence of browning, revealed as seams of lipid-depleted adipocytes (confirmed by the more quantitative analysis by Western blotting; Fig. 3F). B. mRNA was extracted from white adipose tissue from Sdc1−/− and BALB/c mice and the relative expression of differentiation-associated mRNAs was assayed (CD36, FABP4, FASN, LPL, PPARγ). Expression of markers connected with WAT function were not significantly affected in Sdc1−/− mice. (TIF) [file pgen.1004514.s003.tif]

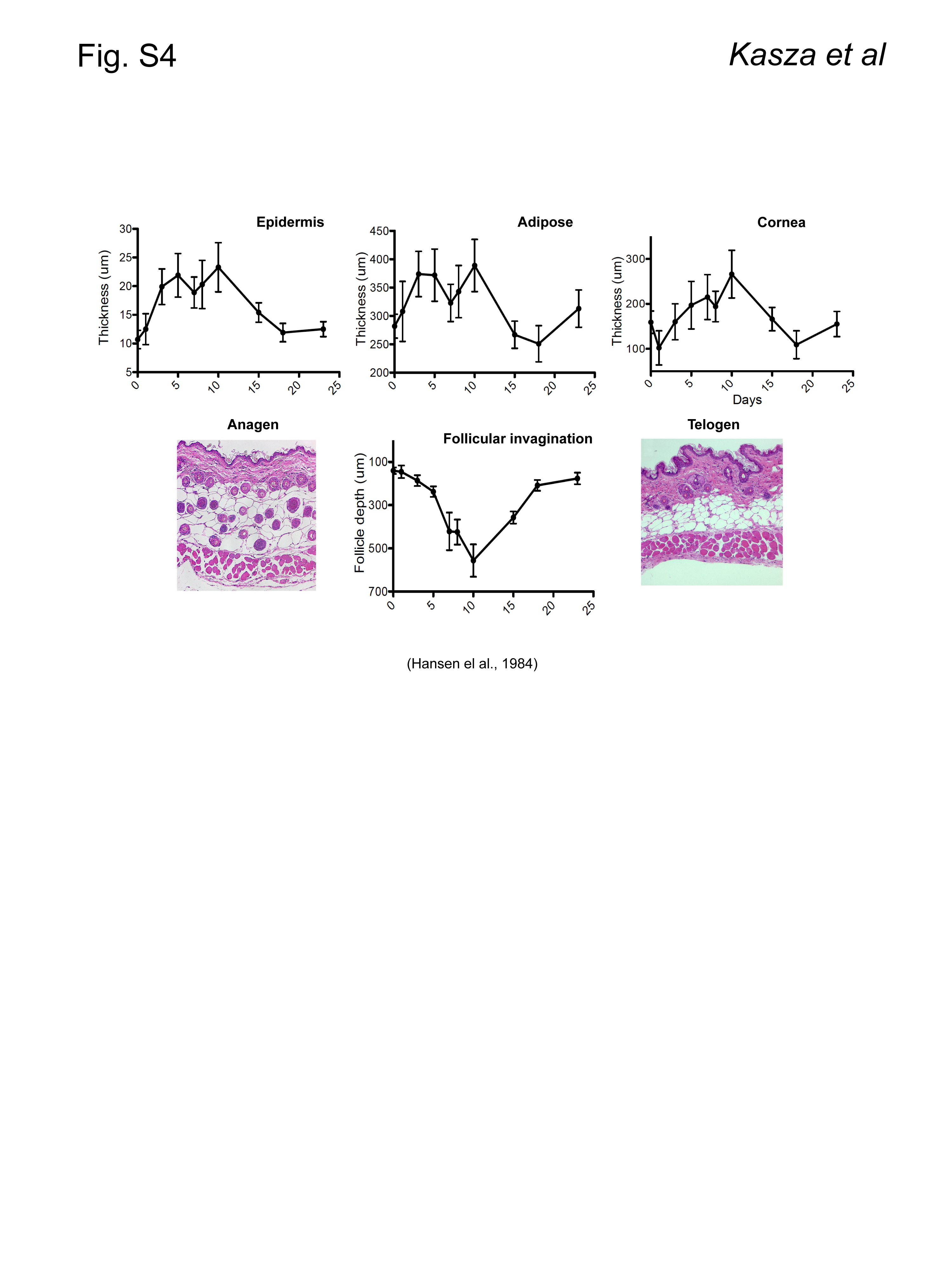

Supplement: Figure S4 — Synergy of hair follicle invagination and expansion of skin-associated tissue layers. Data redrawn from Hansen et al (1984), showing hair regrowth for SAS/4 albino mice, depilated at 11 weeks of age. (TIF) [file pgen.1004514.s004.tif]

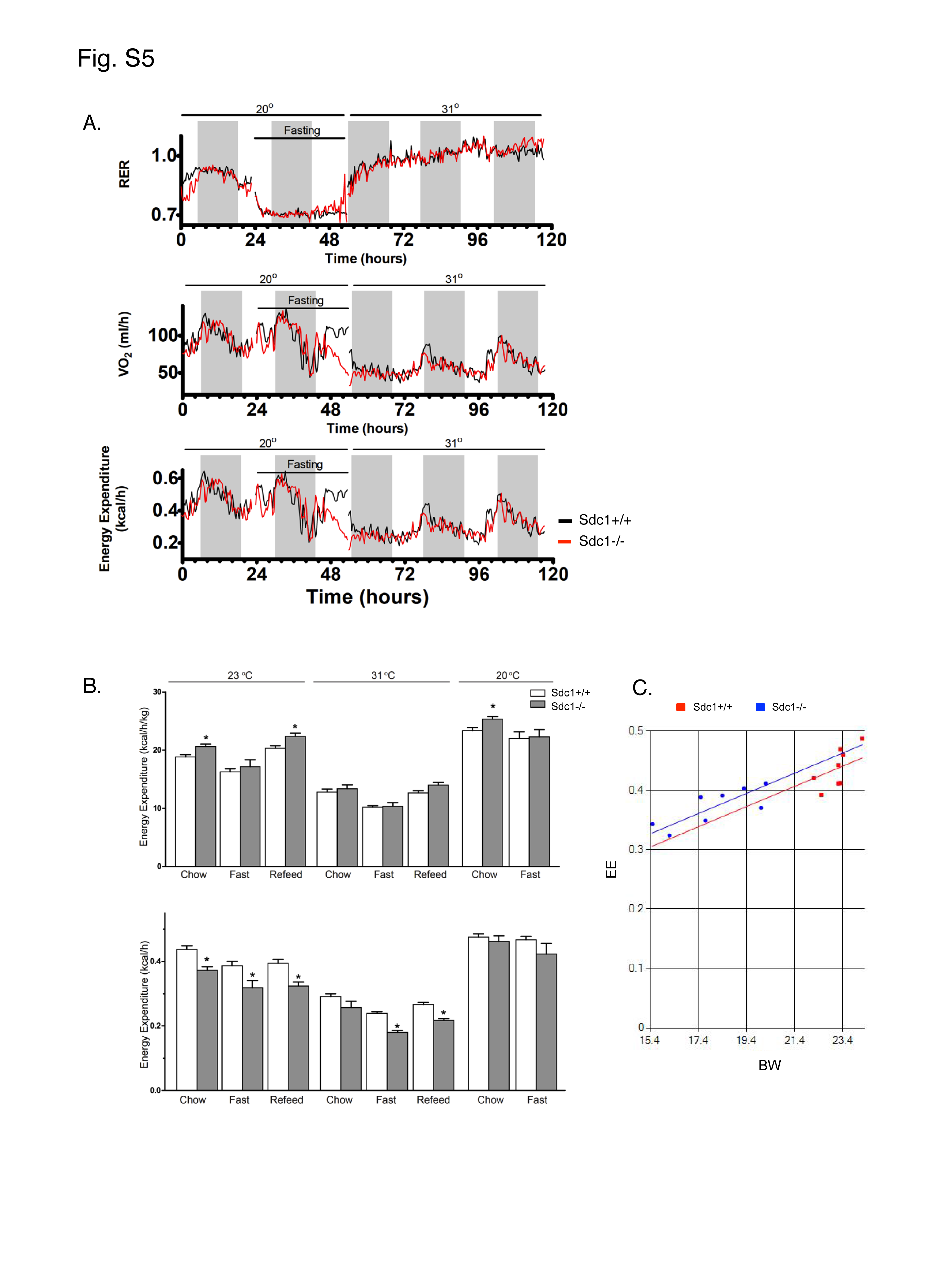

Supplement: Figure S5 — Comparison of metabolic parameters in Sdc1−/− mice housed at various temperatures. A. Timecourse plots from the experiments shown in Figs. 1 and 4 are shown, to compare the RER of BALB/c (black lines; n = 4) and Sdc1−/− mice (red lines; n = 4), and illustrate the VO2 and energy expenditure of mice without body weight corrections. B. Quantitation (energy expenditure, averaged over 48 hours and expressed either per mouse, or corrected for body weights) of metabolic rates in mice housed at various temperatures (20°C, 23°C and 31°C), supplemental to Figs. 1C, D and 4D. C. Multivariate regression analysis of the impact of genotype on energy expenditure. To test whether extra energy expenditure (EE) was observed in Sdc1−/− mice (absent adequate biological insulation), we performed a multivariate regression analysis, using the online tool available at the National Mouse Metabolic Phenotyping Centers (http://www.mmpc.org/shared/regression.aspx). The effect of size and genotype on energy expenditure was compared (n = 8 for each cohort). There was a significant effect of body weight (BW) on EE (as expected given that Sdc1−/− mice are 13% smaller; p = 0.005), but no effect of genotype (p = 0.45). (TIF) [file pgen.1004514.s005.tif]

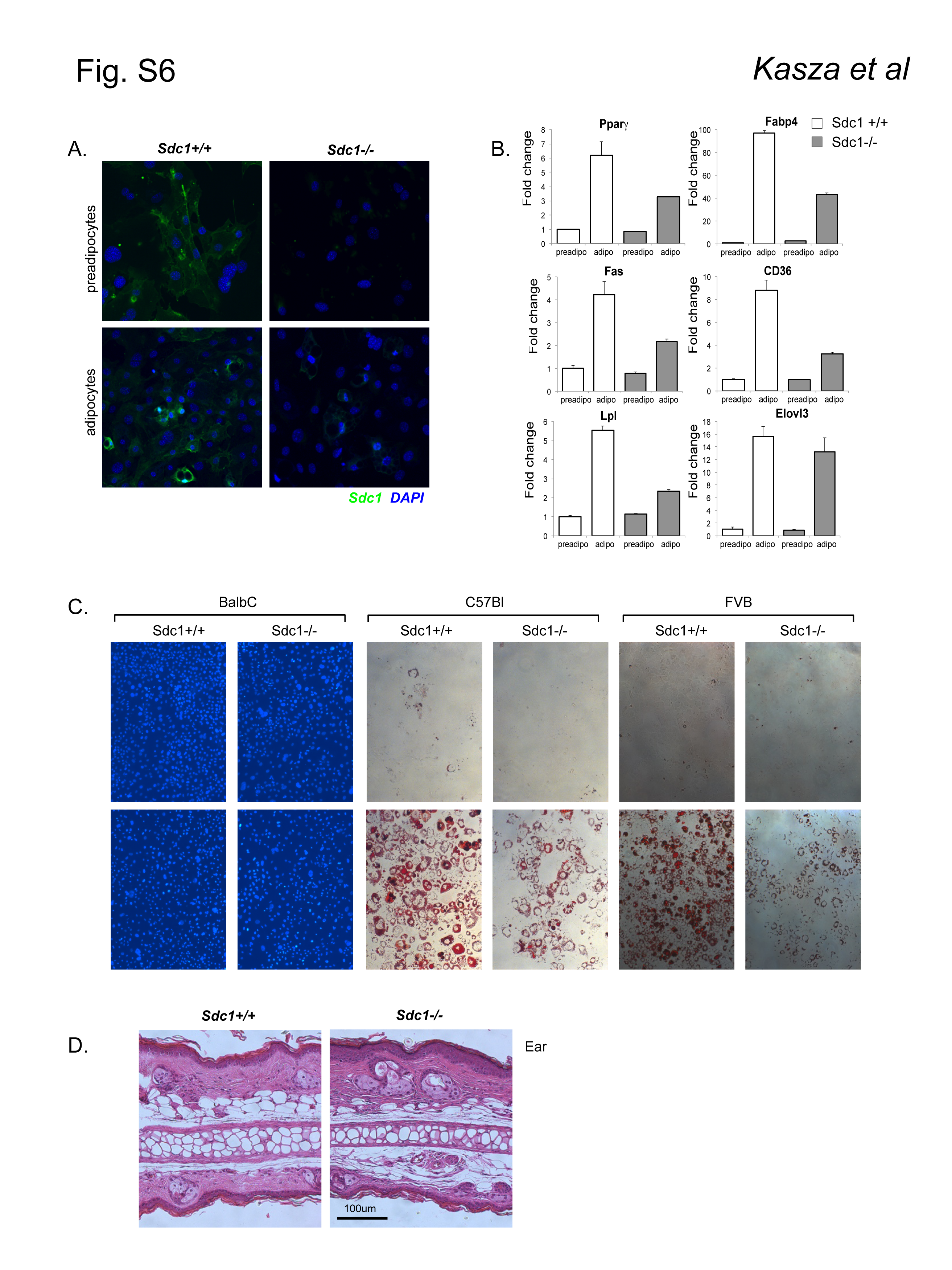

Supplement: Figure S6 — Differentiation of adipocytes in eMSC cultures from Sdc1−/− mice. A. Cultured eMSCs (preadipocytes) were fixed, and immunostained for Sdc1, to show that Sdc1 was expressed in both stages of differentiation, and no Sdc1 was expressed in eMSCs from Sdc1−/− mice. B. Various mRNAs were assayed by qPCR to assess the relative differentiation of Sdc1−/− eMSCs (for comparison with Fig. 5). C. Nuclear stains (Hoecsht) of BALB/c Sdc1−/− and wild type eMSC cultures (to match with Fig. 5D), and from Oil Red-O stained eMSC cultures from C57Bl6 and FVB Sdc1−/− and wild type mice, before (pre-adipocytes, top row) and after induction of differentiation (adipocytes, bottom row). D. The source of eMSCs, the mouse outer ear, is illustrated in an H&E-stained section, showing the central cartilage band and layers of adipocytes on both sides. (TIF) [file pgen.1004514.s006.tif]

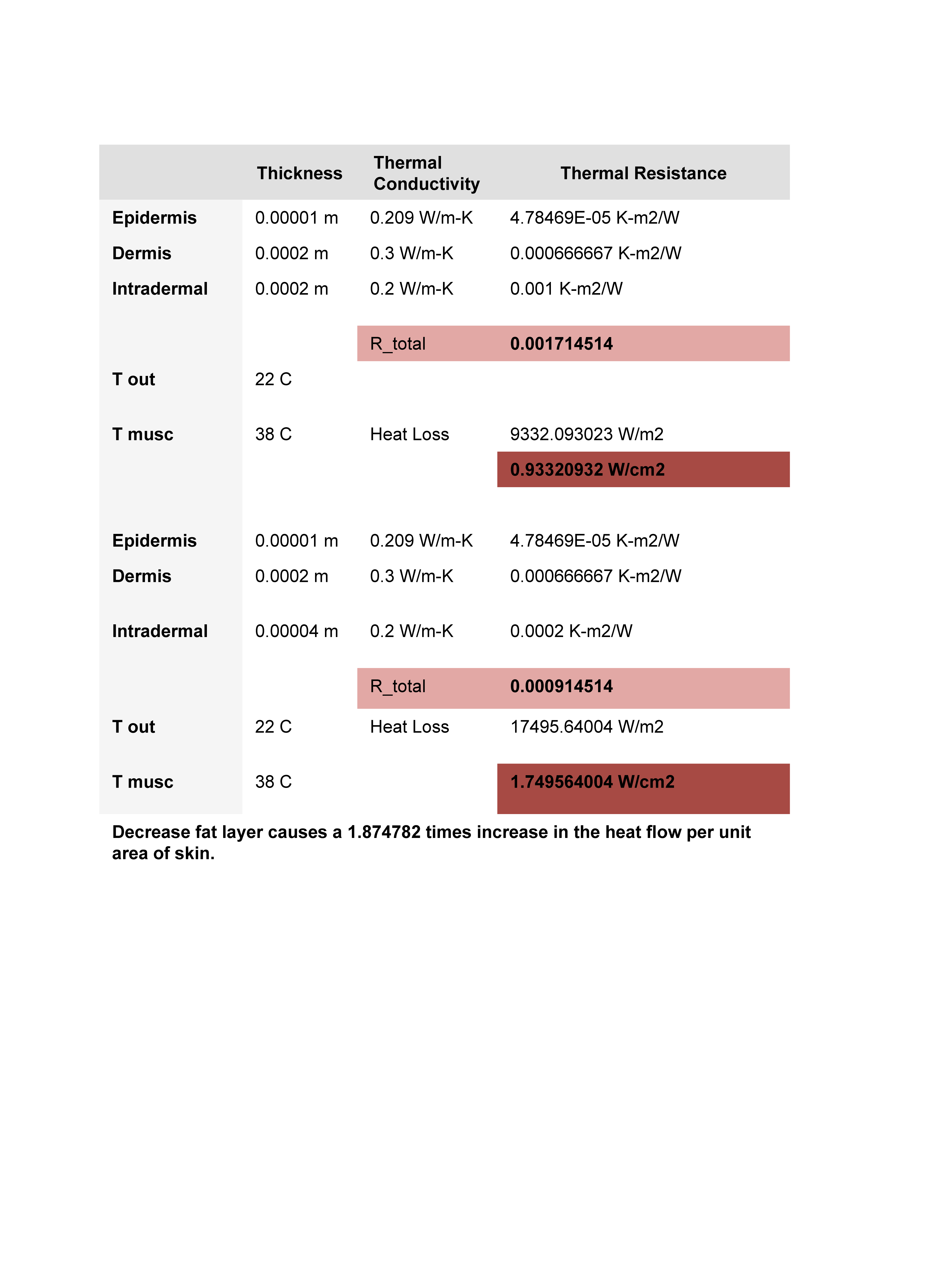

Supplement: Figure S7 — Calculation of thermal conductivity of skins with thin and thicker intradermal fat. The thickness of various layers of skin (dermis, epidermis and intradermal fat; see also Fig. S4), together with their published thermal conductivities, were used to calculate the thermal resistance of 200 µM (top dataset) and 40 µM (bottom dataset) of skin. The result suggests that decreased fat layer could cause a 1.8× increase in heat flow/unit area. (TIF) [file pgen.1004514.s007.tif]
