# Supplementary material for: Syndecan-1 Is Required to Maintain Intradermal Fat and Prevent Cold Stress
Source: PLoS Genet. 2014 Aug 7;10(8):e1004514. doi: 10.1371/journal.pgen.1004514 (PMC4125098; doi:10.1371/journal.pgen.1004514)
Supplement: Text S1 — Details are provided for immunofluorescence staining, microarray processing and qPCR methods. (DOCX) [file pgen.1004514.s009.docx]

**Supplemental Experimental Procedures**

**Immunofluorescence staining**

Skin samples were fixed overnight in 4% paraformaldehyde at 4°C, embedded in paraffin and sectioned. Tissues were deparaffinized, re-hydrated, processed for heat-induced epitope retrieval. After blocking with 10% normal goat serum for 3 h at room temperature samples were incubated overnight with Sdc1 mouse S1ED antibody (gift from Dr. A.C. Rapraeger) and Ki67 proliferation marker (BD Biosciences). Section were rinsed and incubated with anti-mouse IgG-Alexa 488 and anti-rabbit IgG-Alexa 546 secondary antibodies (Invitrogen, Carlsbad, CA) for 1 h at room temperature. Nuclei were stained by mounting in ProLong Gold Antifade Reagent with DAPI (Life Technologies). Sections were visualized on a confocal microscope (BioRad MRC1024).

**Microarray Processing**

For comparison of RNA expression in liver tissues, total RNA was isolated by grinding tissues to a powder in liquid N_2,_ and transferring the powder into the lysis buffer from the RNeasy kit (cat# 74104, Qiagen, Valencia, CA) with the optional DNase protocol from the manufacturer. 5 mgs of each RNA samples was prepared for array hybridization with the Roche cDNA Synthesis Sytem (modified according to the Technical Note July 2011 from Roche Gene Expression), using Roche cDNA Synthesis System (cat# 11117831001), clean up and spin purification. 500 ng of each double strand cDNA was labeled with Cy3-nonamers using the NimbleGen One Color DNA labeling Kit (Roche cat# 06370438001).  Samples were hybridized to the NimbleGen Eukaryotic Gene Expression 12x135K Catalog Array (Roche NimbleGen, cat# 05543797001), representing 44,170 mouse genes from genome build MM9 for each of the 12 arrays.    Data was imported into MeV4.8 [[1](#_ENREF_1)], and fluorescent readings were subjected to log2 transformation followed by median centering. Samples were ordered using a Rank Product Analysis and genes differently expressed in wild type versus *Sdc1-/-* liver samples were identified based on a false discovery rate (FDR) threshold of <10%.

**Quantitative RT-PCR Analysis**

Total RNa was isolated from cells and most tissues using the RNeasy Mini Kit (Qiagen); for fatty tissues the RNeasy Lipid Mini Kit (Qiagen) was used instead. cDNA was generated using a mix of oligo dT and random primers using QuantiTect Reverse Transcription Kit (Qiagen). cDNA (100 ng) was amplified by real time PCR using 5 µL SYBR Green qPCR SuperMix-UDG with Rox (Invitrogen) and 4 µL of forward and reverse primers (0.5 µM). The analysis was performed on each sample in triplicate using an ABI 7900-HT (Applied Biosystems, Foster City, CA). Relative transcript levels were calculated using the comparative Ct method and normalized to appropriate housekeeping genes as follows: succinate dehydrogenase complex, subunit A, flavoprotein (SDHA), tyrosine 3-monooxygenase/tryptophan 5-monooxygenase activation protein, zeta polypeptide (YWHAZ), and hypoxanthine-guanine phosphoribosyltransferase (HPRT) for 3T3-L1 cells; HPRT and YWHAZ for white adipose tissues; phosphoglycerate kinase 1 (PGK1) for liver tissues.

Primer sequences are listed below:

| **Reference Genes** | Forward | exon | Sequence | Reverse | exon | Sequence | Efficiency % |
| --- | --- | --- | --- | --- | --- | --- | --- |
| phosphoglycerate kinase 1 | Pgk1-F | 3 | tgttcccatgcctgacaag | Pgk1-R | 4 | acaggcattctcgacttctg | 100 |
| succinate dehydrogenase complex, subunit A, flavoprotein (Fp) | Sdha-F | 12 | agtgtattgcaagaaggctgtg | Sdha-R | 13 | accaggtctgtgttccaaac | 90 |
| tyrosine 3-monooxygenase/tryptophan 5-monooxygenase activation protein, zeta polypeptide | mhYwhaz-F | 6 | aagacagcacgctaataatgc | mhYwhaz-R | 7 | ttggaaggccggttaattttc | 89-93 |
| hypoxanthine guanine phosphoribosyl transferase 1 | mhHPRT-F | 2 | cctcatggactgattatggacag | mhHPRT-R | 3 | aatccagcaggtcagcaaag | 90-91 |
| **Target Genes** |  |  |  |  |  |  |  |
| CD36 | Cd36-F2 | 12 | gcaaaacgactgcaggtca | Cd36-R | 14 | ttcatcaccaatggtcccag | 80 |
| defensin beta 1 | Defb1-F4 | 1 | atcctctctgcactctgga | Defb1-R | 2 | agacttgtgagaatgccaacac | 81/94 |
| elongation of very long chain fatty acids (FEN1/Elo2, SUR4/Elo3, yeast)-like 3 | Elovl3-F | 3 | tctcagcaaggttgttgaactg | Elovl3-R | 4 | acccgaaggcactttgttc | 82-100 |
| fatty acid binding protein 4, adipocyte | Fabp4-F | 2/3 | aggtgaagagcatcataacccta | Fabp4-R | 4 | gtggaagtcacgcctttcat | 83-89 |
| fatty acid synthase | Fasn-F2 | 38 | ccaagactgactcggctact | Fasn-R4 | 39 | accgagttgagctgggtta | 92-97 |
| fibroblast growth factor 21 | Fgf21-F | 2 | tctttgccaacagccagatg | Fgf21-R | 3 | gggcttcagactggtacac | 92/75 |
| G-protein coupled receptor 12 | Gpr12-F | 2 | gctacatcgcactatgtgactac | Gpr12-R3 | 2 | gtgtaatcggcgatcaaggaat | 79/93 |
| low density lipoprotein receptor | Ldlr-F | 17 | ttcctgtccatcttcttcccta | Ldlr-R | 18 | tggtagactgggttgtcaaagt | 94 |
| lipoprotein lipase | Lpl-F4 | 7 | cgagatttctctgtacggcac | Lpl-R4 | 8 | tccacctccgtgtaaatcaag | 88-94 |
| peroxisome proliferative activated receptor, gamma, coactivator 1 alpha (Pgc1a) | Ppargc1a-F | 15 | gctgtacttttgtggacgga | Ppargc1a-R | 16 | tccagagagtcatacttgctct | 96 |
| peroxisome proliferator activated receptor gamma | Pparg-F3 | 4 | tactgtcggtttcagaagtgc | Pparg-R | 5 | gctggtcgatatcactggag | 92-96 |
| retinoic acid early transcript 1(a,b, c, e) | Raet1(abce)-F |  | gtcagaagaaaccatggccaa | Raet1(abce)-R |  | caagttgcacctaagagagtgtg | 100 |
| syndecan 1 | Sdc1-F | 4 | ggtgcttctcagagccttttg | Sdc1-R | 5 | ccttcttcttcatccggtacag | 95-97 |
| TEA domain family member 2 | Tead2-F2 | 11 | acgctacatgatgaacagtgtc | Tead2-R2 | 12 | ggtggagacttcaaagacgtag | 100/76 |
| uncoupling protein 1 | Ucp1-F | 5 | aactctctgccaggacagt | Ucp1-R | 6 | aacatgatgacgttccaggac | 97 |
| very low density lipoprotein receptor | Vldlr-F | 17 | gtgaccacagcagtatcagaag | Vldlr-R2 | 18 | gccaattcctccacatcaagtag | 95 |

**Reference**

1. Saeed AI, Sharov V, White J, Li J, Liang W, et al. (2003) 4: a free, open-source system for microarray data management and analysis. BioTechniques 34: 374-378.
